# A first-in-class fully modified version of miR-34a with outstanding stability, activity, and anti-tumor efficacy

**DOI:** 10.1038/s41388-023-02801-8

**Published:** 2023-09-05

**Authors:** Ahmed M. Abdelaal, Ikjot S. Sohal, Shreyas Iyer, Kasireddy Sudarshan, Harish Kothandaraman, Nadia A. Lanman, Philip S. Low, Andrea L. Kasinski

**Affiliations:** 1 Department of Biological Sciences, Purdue University, West Lafayette, IN 47907 USA; 2 Department of of Chemistry, Purdue University, West Lafayette, IN 47907 USA; 3 Purdue Institute for Cancer Research, Purdue University, West Lafayette, IN 47907 USA; 4 Department of Comparative Pathobiology, Purdue University, West Lafayette, IN 47907 USA

**Keywords:** Targeted therapies, Nucleic-acid therapeutics

## Abstract

Altered by defects in p53, epigenetic silencing, and genomic loss, the microRNA miR-34a represents one of the most clinically relevant tumor-suppressive microRNAs. Without question, a striking number of patients with cancer would benefit from miR-34a replacement, if poor miR-34a stability, non-specific delivery, and delivery-associated toxicity could be overcome. Here, we highlight a fully modified version of miR-34a (FM-miR-34a) that overcomes these hurdles when conjugated to a synthetically simplistic ligand. FM-miR-34a is orders of magnitude more stable than a partially modified version, without compromising its activity, leading to stronger repression of a greater number of miR-34a targets. FM-miR-34a potently inhibited proliferation and invasion, and induced sustained downregulation of endogenous target genes for >120 h following in vivo delivery. In vivo targeting was achieved through conjugating FM-miR-34a to folate (FM-FolamiR-34a), which inhibited tumor growth leading to complete cures in some mice. These results have the ability to revitalize miR-34a as an anti-cancer agent, providing a strong rationale for clinical testing.

## Introduction

Reduced microRNA (miRNA) stability, potential immunogenic effects related to unmodified RNAs, and lack of safe delivery vehicles are major drawbacks associated with transitioning miRNAs into the clinic [1–3]. Unmodified miRNAs are rapidly degraded by nucleases which hinders their activity, demands the use of high and repetitive dosing, and makes them incompatible with in vivo applications [3]. Several chemical modifications have been used to stabilize RNAs, including 2′-O-methyl and 2′-fluoro modifications to the ribose, and phosphorothioate substitutions to the backbone [3–5]. The ribose modifications improve binding affinity and provide protection against nucleases while the phosphorothioate bonds confer additional resistance to exonucleases [4]. The 2′-O-methyl modification has also been used to avoid immune system stimulation that is triggered by the delivered RNA. Nonetheless, extensive modification could impede the silencing activity of miRNAs by altering target-gene affinity and through increasing the stability of the miRNA duplex making it difficult for RISC to unwind the strands and selectively load the active strand [5]. Thus, it is important to carefully design and select modifications that enhance miRNA stability, but at the same time modification patterns that are suitable for RISC loading and target gene repression. In the case of siRNAs and antisense oligonucleotides (ASOs), chemical modifications are effective, prolonging target gene silencing, ultimately reducing the therapeutic doses [4, 6–8]. However, the same benefit has yet to be harnessed for miRNA duplexes, RNAs that have the capacity to function as multi-drug cocktails, targeting cohorts of relevant genes.

MiRNAs are short noncoding RNAs that have the unique ability to downregulate multiple genes at the same time [9–11]. For example, in the case of tumor suppressive miRNAs, several oncogenic pathways that control cell proliferation, migration and invasion, resistance to apoptosis, and immune evasion can all be regulated by miRNA-34a, which targets the Androgen receptor (AR), C-MYC, AXL, MET, SIRT1, CD44, PDL-1, and others [12–18]. Despite the great benefit achieved through targeting multiple genes simultaneously [19, 20], predicting the effect of chemical modification on the pleotropic targets of miRNAs is difficult. Thus, studies that laboriously evaluate the impact of chemical modifications on miRNA stability and activity are needed to advance modified miRNA therapeutics into the clinic.

While enhanced stability is critical for advancing miRNA-based therapeutics into human patients, equally important is achieving specific targeting to the correct tissue in the absence of toxicity. Recent methods to accomplish this include use of ligands to deliver the RNA [3, 21]. This approach relies on conjugating an RNA to a targeting ligand that has high affinity and specificity for a receptor that is upregulated by the targeted cells. Indeed, many tumors upregulate cell surface receptors. Ligands that bind to these receptors, if engineered to carry an anti-tumor warhead, can target drugs specifically to tumor cells [22]. For a receptor to be useful for delivery, the target receptor must meet two criteria: it must be overexpressed on the cancer cell relative to normal cells, and the level of expression must be sufficient to enable delivery of therapeutic quantities. The folate receptor (FR) fulfills both of these criteria, where it is overexpressed in breast, lung, ovarian, colorectal, and other cancers. Examples of using FR for delivery of imaging and therapeutic agents to these tumors are highlighted in our work using folate-NIR conjugates for image-guided surgical resection that gained FDA approval in 2021 and studies using folate-miRNAs targeted to FR overexpressing tumors [21, 23–25]. Nonetheless, RNA activity is limited by entrapment of the conjugate in the endosomes and RNA degradation by various nucleases. Although inclusion of various endosomal escape moieties can increase cytosolic accumulation of ligand-conjugated RNAs [26, 27], the presence of cellular nucleases reduces the half-life, concealing the full potential of these RNAs. One way to enhance RNA stability is through the use of modified nucleotides. However, the direct impact of full chemical modification on the activity of tumor suppressive miRNA duplexes, how these modifications affect targeting, and the efficacy of fully modified miRNAs in vivo are not well understood.

To overcome the aforementioned challenges and to expand our understanding of the effect of chemical modifications on miRNA duplex activity, we developed the first chemically modified miR-34a duplex and compared its stability and activity to a partially modified version of miR-34a, a version with modifications akin to commercially available miR-34a mimics [28]. Effects of fully modified miR-34a were evaluated in cells in culture and in vivo following transfection or conjugation to folate in preparation for clinical advancement. Indeed, small RNAs have tremendous power to downregulate the expression of genes that cancer cells are addicted to, and they can do so with limited toxicity, if designed appropriately. Proper design requires mechanisms to reduce toxicity, increase specificity, facilitate correct intracellular distribution, and increase RNA stability. Here we present the first significant success in all of these areas for treating oncological disease.

## Results

### Design, synthesis, and in vitro serum stability of partially and fully modified miR-34a

**Fig. 1:**
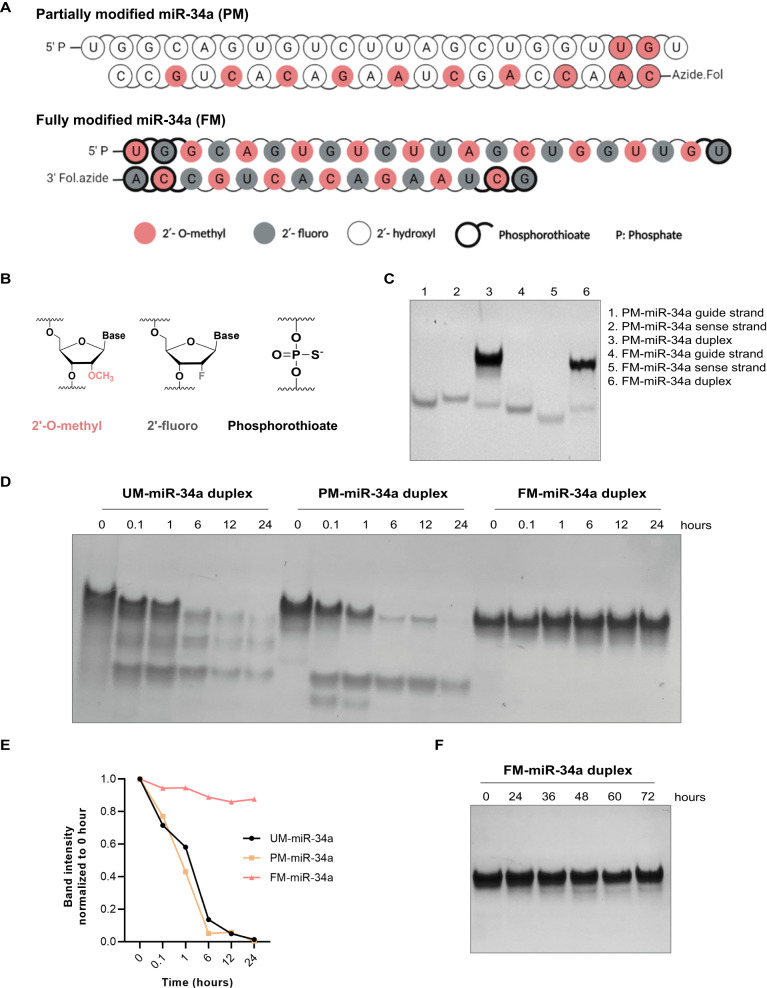
Chemical composition and stability of partially modified (PM) and fully modified (FM) miR-34a. **A** Chemical modification pattern of PM-miR-34a and FM-miR-34a. **B** Structure of the various chemical modifications used in (**A**). **C** Representative gel-Red-stained poly-acrylamide gel of PM- and FM-miR-34a highlighting successful annealing of miRNA duplexes as indicated by mobility shifts on the gel (*n* > 5). **D** Representative gel-Red-stained poly-acrylamide gel of unmodified (UM-), PM- and FM-miR-34a folloiwng exposure to 50% serum over a time course (*n* = 3). **E** Band intensities from (**D**) normalized to 0 h. **F** Incubation of FM-miR-34a with 50% serum for the indicated times followed by resolving on a poly-acrylamide gel and staining with gel-Red (representitive image shown from *n* = 3).

When designing a modified RNA oligonucleotide for modulating gene expression, it is necessary to ensure that the incorporated modifications do not interfere with gene silencing. Previously, we synthesized miR-34a containing a minimal number of 2′-O-methyl modifications to the ribose sugars, which is similar in chemical composition to commercially available miR-34a mimics [28] -we refer to this as partially modified miR-34a (PM-miR-34a) [21]. To understand the impact of full chemical modification on miRNA stability and activity, we designed a fully modified miR-34a (FM-miR-34a) in an asymmetric pattern that contains a 22-nucleotide guide strand annealed to a 15-nucleotide complementary strand, which lowers the thermokinetics between the two strands facilitating strand displacement by the RNA Induced Silencing Complex (RISC), a similar pattern used to stabilize siRNA [4]. Each strand contains an alternating pattern of 2′-O-methyl and 2′-fluoro modified sugars and phosphorothioate linkages at the 3′ and 5′ ends of each strand to reduce immunogenicity and provide exonucleases resistance (Fig. 1A, B). Both PM-miR-34a and FM-miR-34a duplexes were generated and confirmed (Fig. 1C). The stability of FM-miR-34a was compared to the stability of PM-miR-34a and unmodified miR-34a duplexes following incubation in 50% serum over a time course. While unmodified and PM-miR-34a were destabilized rapidly following exposure to serum, FM-miR-34a was completely resistant up to 24 h and remained intact for at least 72 h (Fig. 1D–F).

### Comparing gene targeting of FM-miR-34a to PM-miR-34a

**Fig. 2:**
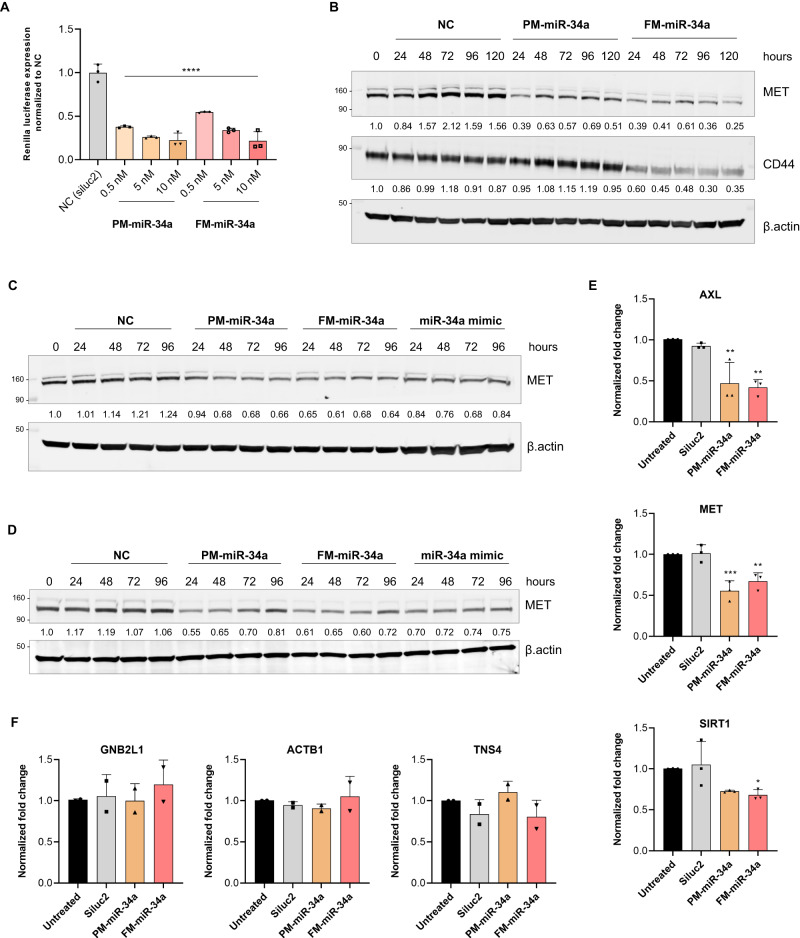
Comparison of cellular activity of PM and FM-miR-34a. **A** Silencing of miR-34a Renilla sensor 72-hours post-transfection of MB-231-miR-34a sensor cells with PM or FM-miR-34a duplexes (*n* = 3, 24 and 48 h time point data can be found in Fg S1A). **B** Representative immunoblot indicating reductions of MET and CD44 post-transfection of MB-231 cells with 50 nM FM-miR-34a or PM-miR-34a for the times indicated. Protein intensity was normalized to β.actin and represented as a fold change relative to 0 h. (*n* = 3) **C, D** Representative immunoblot images highlighting reduction of MET expression post-transfection of Hela and IGROV1 cells with 50 nM FM-miR-34a, PM-miR-34a, or miR-34a mimic for the times indicated. Protein intensity was normalized to β.actin and represented as a fold change relative to 0 h. (*n* = 3) **E,** **F**, Evaluation of the expression of endogenous miR-34a targets or non-targets by qRT-PCR from MB-231 cells following transfection with 50 nM PM-miR-34a or FM-miR-34a (error bars: means ± SD, *n* = 3 biological replicates in case of **E** and *n* = 2 in case of **F**). All experiments were performed using three technical replicates per treatment. ^*^p < 0.05, ^**^p < 0.01, ^***^p < 0.001, ^****^p < 0.0001, relative to siluc2, one-way Anova with Dunnett’s multiple comparison test against NC (Siluc2). GAPDH was used as an endogenous control for all qRT-PCR experiments.

To evaluate the effect that the chemical modifications have on miR-34a function, we compared the silencing activity of FM-miR-34a to PM-miR-34a on a synthetic target with a 100% complementary sequence and on endogenous biological targets of miR-34a. Effects on the synthetic target were conducted in MB-231 cells engineered to stably express a miR-34a complementary sequence downstream of the Renilla gene (MB-231-miR-34a sensor cells). Transfection of MB-231-miR-34a sensor cells with FM-miR-34a or PM-miR-34a significantly downregulated Renilla luciferase expression suggesting that the various chemical modifications do not interfere with miR-34a silencing activity (Fig. 2A and Fig. S1A). Similarly, both PM- and FM-miR-34a duplexes downregulated an additional reporter with a miR-34a target sequence downstream of firefly luciferase following transient transfection (Fig. S1B). The silencing activity of endogenous miR-34a targets was also evaluated in MB-231 (breast), Hela (cervical), IGROV1 (ovarian), and LNCaP (prostate) cancer cell lines. In all cases transfection of FM-miR-34a resulted in a similar, or more prominent downregulation of miR-34a target proteins, including MET, CD44, and androgen receptor (AR) (Fig. 2B–D, and Fig. S1C). FM-miR-34a and PM-miR-34a also significantly downregulated miR-34a target mRNAs *AXL* and *MET*, and FM-miR-34a significantly downregulated *SIRT1* (Fig. 2E), while neither miR-34a duplex altered the levels of transcripts not predicted to be miR-34a targets (Fig. 2F). These results indicate that the proposed full chemical modifications, when applied to miR-34a, results in a similar or enhanced silencing of select miR-34a target genes, which propelled us to compare the entire transcriptome between cells transfected with either PM-miR-34a or FM-miR-34a.

### Transcriptomic comparison of PM-miR-34a and FM-miR-34a targeting

**Fig. 3:**
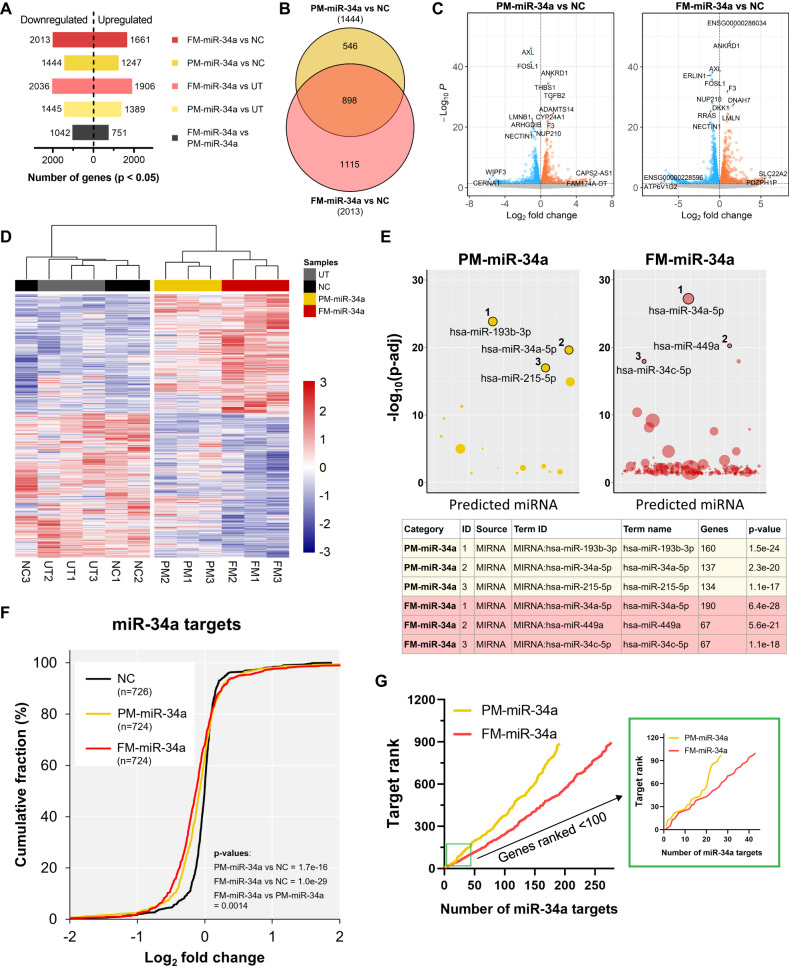
FM-miR-34a gene targeting is more robust than PM-miR-34a. **A** Overall number of statistically significant (*p* < 0.05) differentially expressed genes in MB-231 cells transfected with either PM-miR-34a or FM-miR-34a in comparison to siluc2-transfected (NC) or untreated (UT) cells; *p*-values calculated by Wald Chi-Squared Test in *DESeq2* package. **B** Overlap of statistically significant downregulated genes in PM-miR-34a vs. NC and FM-miR-34a vs. NC comparisons (a similar analysis for upregulated genes can be found in fig S2A). **C** Volcano plots of up-regulated and down-regulated genes compared between cells transfected with PM-miR-34a and NC, or between FM-miR-34a and NC. Gene labels represent the top 6 up-and down-regulated genes based on lowest *p*-value and top 2 up- and down-regulated genes based on fold-change from each comparison. Dashed line represents *p*-value cut-off (0.05). Grey dots indicate non-significant genes. **D** Clustering heatmap of differentially expressed genes sorted based on the most differentially regulated between the FM-miR-34a and NC; distance method = “Euclidean”, clustering method = “ward.D2”. **E** Unbiased miRNA target enrichment analysis of statistically significant downregulated genes comparing PM-miR-34a to NC and FM-miR-34a to NC based on mirTarBase database. Dot size represent the number of target genes identified that are predicted to be targeted by the miRNA indicated. From each comparison, the top 3 predicted miRNAs are labelled and their *p*-value, and the number of target genes identified from the experimental data are indicated in the table. **F** Cumulative plot demonstrating the effect of transfecting NC, PM-miR-34a, or FM-miR-34a on log_2_ fold-change distributions of transcripts identified as known/predicted miR-34a targets (miRDB database). Numbers in brackets represents the number of mRNA transcripts identified in each set that overlap with known/predicted miR-34a targets in miRDB database. The log_2_ fold-change values are relative to UT. For representative p-values, two cumulative plots were compared, and p-values were calculated using Two-sample Kolmogorov-Smirnov test. **G**, Line plot indicating cumulative number of downregulated genes in PM-miR-34a vs. NC (yellow) or FM-miR-34a vs. NC (red) that overlap with known/predicted miR-34a targets in miRDB database and their rank. Target rank (y-axis) starts with the most highly ranked miR-34a target genes to the less stringent targets. Inset shows a similar plot for the top 100-ranked miR-34a targets.

In order to compare the global transcript profile following FM-miR-34a or PM-miR-34a exposure, and to evaluate if FM-miR-34a is behaving as endogenous miR-34a, MB-231 cells were transfected with either PM-miR-34a or FM-miR-34a, RNAseq was conducted, and gene expression and associated biological processes and pathways were evaluated. Generally, gene expression changes in cells transfected with FM-miR-34a mirrored expression changes in PM-miR-34a transfected cells, albeit targeting by FM-miR-34a was more robust (Fig. 3A). Of all the genes altered by PM-miR-34a, 62.2% of the downregulated genes (Fig. 3B) and 59.8% of the upregulated genes (Fig. S2A) were also significantly altered by FM-miR-34a. Of the downregulated genes, the miR-34a target *AXL* had the lowest p-value following either PM- or FM-miR-34a transfection (see Fig. 3C). Clustering analysis further supported the targeting similarities by PM-miR-34a and FM-miR-34a transfected cells, which grouped together, away from untransfected or negative control transfected cells (Fig. 3D). Similarities were also highlighted in gene ontology terms using either the downregulated (Fig. S2B) or upregulated (Fig. S2C) gene-sets from PM-miR-34a and FM-miR-34a datasets. While similarities were evident, the heatmap also indicated that transcripts in FM-miR-34a transfected cells were often more robustly altered in expression relative to PM-miR-34a transfected cells.

Although the above analyses highlight similarities between the two miRNAs at a global level, it was crucial to determine if FM-miR-34a was indeed mimicking the targeting activity of endogenous miR-34a. To evaluate this, we conducted an unbiased miRNA target enrichment analysis to determine which miRNA is most likely to target the significantly downregulated genes in the FM-miR-34a or PM-miR-34a datasets, with the expectation that miR-34a-5p should be the top predicted miRNA for both datasets. Indeed, miR-34a-5p was the top predicted miRNA for the FM-miR-34a gene-set (*p*-value = 6.4e-28) followed by miR-449a and miR-34c-5p – all members of the miR-34 family (Fig. 3E), suggesting a high level of similarity to endogenous miR-34a activity. However, for the RNAs downregulated in the PM-miR-34a dataset, miR-193b-3p was the top predicted miRNA (*p*-value = 1.5e-24), followed by miR-34a-5p (*p*-value = 2.3e-20), and miR-215-5p (Fig. 3E). The robust targeting of miR-34a target genes by FM-miR-34a was highlighted by the increased number of downregulated miR-34a targets in the FM-miR-34a gene-set in comparison to the PM-miR-34a gene-set (190 vs 137) (Table S1). To further validate our findings, we expanded our analysis to all miR-34a targets, including ones not significantly altered in our data-sets, and determined the global effect of PM-miR-34a and FM-miR-34a on the cumulative log_2_ fold-change distribution of all known/predicted miR-34a targets (curated from miRDB). Both PM-miR-34a and FM-miR-34a significantly altered the cumulative distribution in comparison to NC, indicating downregulation of miR-34a targets; however, repression by FM-miR-34a was often greater, leading to a significant difference in cumulative distribution between PM-miR-34a and FM-miR-34a (Fig. 3F). Finally, we obtained the rankings for the curated list of miR-34a targets from the miRDB database and distributed genes by target ranking on the y-axis – low ranked targets have stronger data supporting that miR-34a is indeed targeting them. Consistent with previous results, FM-miR-34a not only had a larger number of miR-34a targets repressed than PM-miR-34a transfected cells (277 vs 191), but transfection with FM-miR-34a also resulted in a more robust downregulation of the targets (Fig. 3G, Fig. S3 and Table S2). Among the top 100 ranked miR-34a predicted targets, FM-miR-34a downregulated 43% whereas PM-miR-34a downregulated only 27% of them (Fig. 3G, inset). While the contribution of the passenger strand was anticipated to be negligible, we conducted a similar analysis on the predicted targets of miR-34a-3p and found that ~5 and 9% of miR-34a-3p targets were altered by PM- and FM-miR-34a, respectively (Fig. S3C). Collectively, these findings indicate that FM-miR-34a downregulates more miR-34a targets and causes more robust downregulation in comparison to PM-miR-34a.

### FM-miR-34a inhibits cancer cells proliferation, migration, and invasion in-vitro

**Fig. 4:**
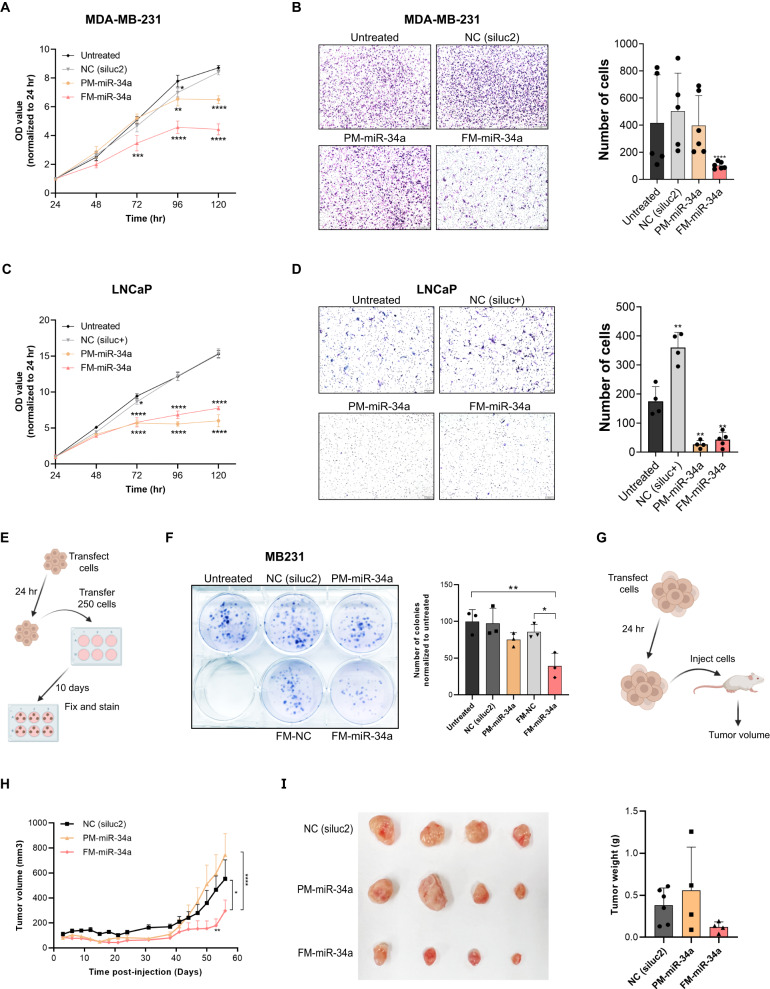
FM-miR-34a inhibits cancer cells proliferation, migration, and invasion. **A**, **C** Effect of PM-miR-34a or FM-miR-34a on proliferation of MB-231 cells or LNCaP cells), measured by SRB assay. Graphs depict one representative experiment (*n* = 6 technical replicates) from a total of three biological replicates, error bars: mean ± SD, ^*^p < 0.05, ^**^p < 0.01, ^***^p < 0.001, ^****^p < 0.0001, one-way Anova with Dunnett’s multiple comparison test against untreated. **B** Representative images (left) and quantification (right) of MB-231 cells that migrated through a 6μm-pore size transwell over 6 h following transfection with PM-miR-34a or FM-miR-34a for 72 h (*n* = 3). **D** Representative images (left) and quantification (right) of LNCaP cells that invaded through a matrix over 96 hr following transfection with PM-miR-34a or FM-miR-34a for 48 h (*n* = 3). **E** Schematic of the colony formation assay. **F** Evaluation of the colony forming potential of cells transfected with PM-miR-34a, FM-miR-34a, or the respective controls. Representative image (left side) and quantifcication of the number of colonies normalized to untreated (right side). Error bars: Mean ± SD. ^*^p < 0.05, ^**^p < 0.01, one-way ANOVA with Tukey’s multiple comparisons test. (*n* = 3) **G**–**I**, Subcutaneous tumor growth of MB-231 cells transfected with 50 nM PM-miR-34a, FM-miR-34a, or NC duplexes. **G** Schematic showing the steps involved in the procedure. **H** Tumor volume over the course of the study. Cells were implanted on both left and right flanks of each mouse (total tumors *n* = 6 for NC and *n* = 4 for PM-miR-34a and FM-miR-34a). Error bars: mean ± SEM (only error bars above the lines are shown); ^*^p < 0.05, ^**^p < 0.01, ^****^p < 0.0001; two-way ANOVA with Tukey’s multiple comparisons test. **I** Image (left) and weights (right) of the tumors harvested from the mice at the end of the study, error bars: mean ± SD.

Based on the outstanding targeting of FM-miR-34a, we hypothesized that FM-miR-34a would more significantly impact the phenotypes of cells transfected with FM-miR-34a. To address this hypothesis, the impact of full chemical modification of miR-34a on cancer cell proliferation, migration, and invasion was determined. In comparison to PM-miR-34a, transfecting MB-231 cells with FM-miR-34a resulted in a significant and stronger reduction in cell proliferation (Fig. 4A) and inhibition of migration (Fig. 4B). In LNCaP cells, both FM-miR-34a and PM-miR-34a significantly inhibited cell proliferation (Fig. 4C) and invasion (Fig. 4D) to a similar level. These results are consistent with the effect of FM-miR-34a and PM-miR-34a on target genes (see Fig. 2B), where for CD44, FM-miR-34a produced a much stronger downregulation in MB-231 cells. Importantly, proliferation of non-tumorigenic BEAS2B cells was not altered in the presence of either miR-34a duplex, suggesting that the effect on cancer cells might be the result of addicted oncogenic signaling that is suppressed by miR-34a (Fig. S4). To further compare the effect between FM-miR-34a and PM-miR-34a on MB-231 cell proliferation, a clonogenic assay following transfection was performed. This assay allowed us to determine the effect on cell proliferation over a longer time course, which we hypothesized would be greater for FM-miR-34a due to its enhanced stability. Indeed, FM-miR-34a induced a significant inhibition of MB-231 clonogenic capacity, as indicated by smaller colonies and overall reduced number of colonies (Fig. 4E, F).

To determine the effect of FM-miR-34a on tumor growth and development, MB-231 cells transfected with FM-miR-34a or PM-miR-34a were implanted into immunodeficient mice. Tumor development of cells transfected with FM-miR-34a was significantly delayed in comparison to cells transfected with PM-miR-34a (Fig. 4G, H). And, despite being statistically insignificant, tumors harvested from the FM-miR-34a group were generally smaller than tumors harvested from the PM-miR-34a group, with a mean tumor weight of 0.12 g versus 0.55 g respectively (Fig. 4I). Overall, these results indicate that FM-miR-34a induces a similar, or enhanced reduction in proliferation, migration, and invasion of cancer cells in comparison to PM-miR-34a.

### FM-miR-34a activity is dependent on loading into Argonaute (Ago)

**Fig. 5:**
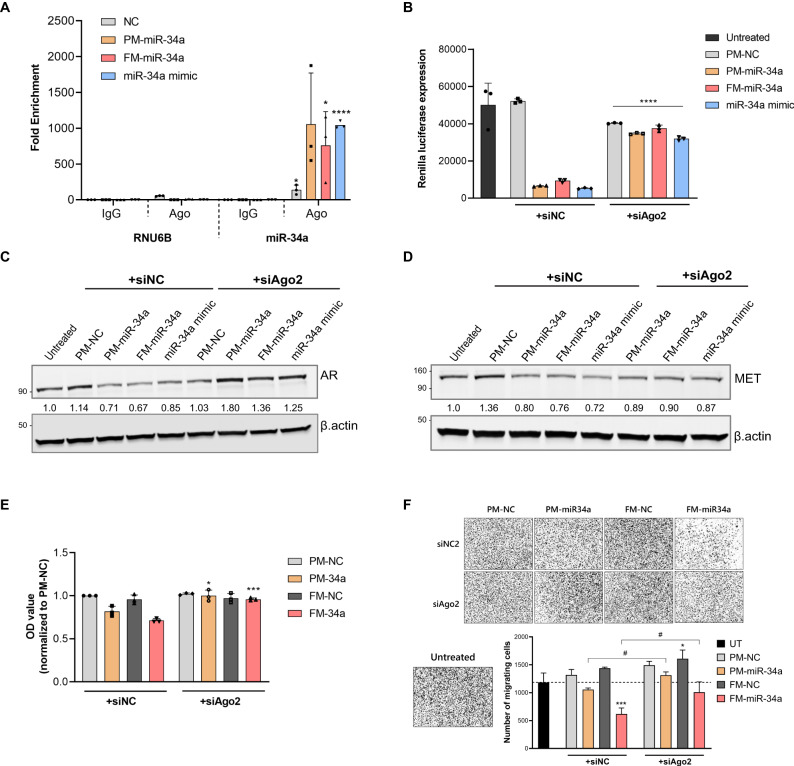
FM-miR-34a activity requires AGO loading. **A** Quantification of miR-34a using qRT-PCR following Ago immunoprecipitation. MB-231 cells were transfected with NC, PM-miR-34a, FM-miR-34a, or a commercial miR-34a mimic followed by Ago immunoprecipitation and quantification of miR-34a. miR-34a expression in Ago-IP samples was normalized to miR-34a in IgG-IP and input (representative experiment of *n* = 3). Significance was determined against IgG-IP samples. **B**, **D** Effect of Ago2 knockdown on miR-34a activity indicating the contribution of Ago2 to FM-miR-34a activity. **B** Renilla luciferase expression in MB-231-miR-34a sensor cells following transfection with NC, PM-miR-34a, FM-miR-34a, or the miR-34a mimic in the presence or absence of siRNA targeting Ago2 (*n* = 3 biological replicates). **C,** **D** Representative Western blot images depicting derepression of Androgen Receptor AR, or MET expression following transfection of LNCaP or MB-231 cells, respectively with 50 nM PM-miR-34a or FM-miR-34a in the presence of 50 nM siAgo2 (*n* = 3). Protein intensity was normalized to β.actin and represented as a fold change relative to untreated. **E** Proliferation of MB-231 cells measured 120 h following transfection with 50 nM PM-miR-34a or FM-miR-34a in the presence or absence of 50 nM siAgo2. One representative biological experiment of three is shown, including *n* = 3 technical replicates. **F** Migration of MB-231 cells measured by migration assay following transfection with 50 nM PM-miR-34a or FM-miR-34a in presence of 50 nM siAgo2 (error bars: mean ± SD, *n* = 2, ^*^p < 0.05, ^***^p < 0.001, Dunnett’s one-way ANOVA compared to UT (untreated); ^#^*p* < 0.05, two-tailed Student’s *t-*test). For panels **A**, **B** and **E**, error bars: Mean ± SD. ^*^p < 0.05, ^**^p < 0.01, ^***^p < 0.001, ^****^p < 0.0001, two-tailed Student’s *t* test. Significance was determined against IgG-IP in **A**, and against the same treatment in the presence of the siNC in **B** and **E**.

There are multiple mechanisms by which synthetic oligonucleotides could reduce the expression of target genes independently of the miRNA-mediated pathway. For example, antisense oligonucleotides can downregulate target genes by triggering RNase H-mediated degradation or by steric hindrance [29]. To validate that FM-miR-34a functions using the same machinery as endogenous miR-34a, the necessity of Argonaute (Ago), the major component of RISC that is essential for endogenous miRNA activity, was assessed. RNA immunoprecipitation was performed following transfection of MB-231 cells with FM-miR-34a, PM-miR-34a, a commercial miR-34a mimic that is similar in chemistry to PM-miR-34a, or NC. Ago-loaded RNA was immunoprecipitated with an anti-Ago antibody followed by miR-34a quantification. Subsequent analysis revealed that FM-miR-34a, PM-miR-34a, and the miR-34a mimic were efficiently loaded into Ago, indicating use of similar targeting machinery as endogenous miRNAs (Fig. 5A, Fig. S5A). To further confirm the role of Ago in FM-miR-34a activity, Ago2 was knocked down and the effect of FM-miR-34a silencing of the Renilla reporter or endogenous miR-34a genes was evaluated. The synthetic reporter and endogenous targets were all de-repressed when FM-miR-34a was combined with Ago2 knockdown (Fig. 5B–D, Fig. S5B). Additionally, the inhibitory effect of FM-miR-34a on proliferation and migration of MB-231 was lost when FM-miR-34a was combined with Ago2 silencing (Fig. 5E, F). Overall, these results indicate that FM-miR-34a loading into Ago2 mediates target gene silencing of both exogenous as well as endogenous targets leading to phenotypic effects.

### Evaluation of FM-FolamiR-34a activity in vivo

**Fig. 6:**
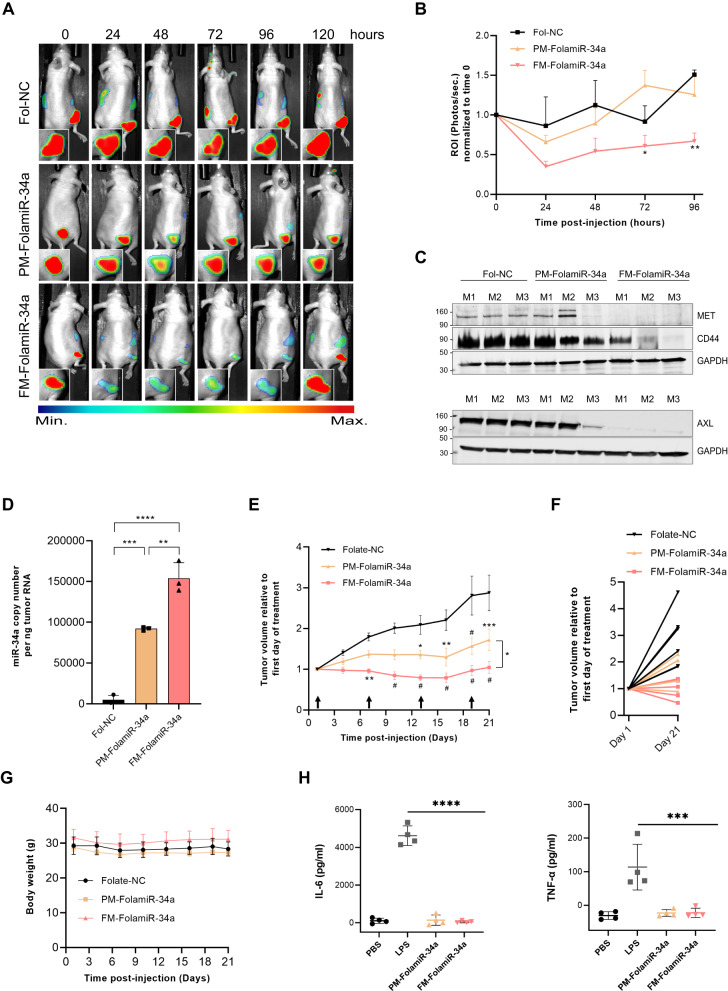
In vivo efficacy of FM-FolamiR-34a. **A** Representative image of Renilla luciferase sensor signal in nude mice implanted with MB-231-miR-34a sensor cells following intravenous injection of a single 1.5 nmol dose of folate-NC (siluc2), PM-FolamiR-34a, or FM-FolamiR-34a. **B** Effect of FM-FolamiR-34a delivery on the miR-34a-Renilla sensor signal over 96 h. Data normalized to day 0; error bars: means ± SEM (only error bars above each data point are shown), *n* = 3 mice per group. ^*^p-value 0.05 between PM- and FM-FolamiR-34a, ^**^p-value < 0.01 between FM-FolamiR-34a and Fol-NC. **C** Immunoblot image of miR-34a targets (MET, CD44, and AXL) in excised MB-231 tumors 120 h after intravenous injection with a single 1.5 nmol dose of folate-NC (siluc2), PM-FolamiR-34a, or FM-FolamiR-34a duplexes, M: Mouse. (*n* = 3 mice per treatment) **D** miR-34a levels from excised MB-231 tumors quantified by qRT-PCR 120 h post-injection with the various folate conjugates (*n* = 3 mice, with at least 3 technical replicates; error bars: means ± SD; one-way ANOVA with Tukey’s multiple comparisons test). **E** Tumor volumes following treatment with the various folate-miRNA conjugates (folate-NC: *n* = 6 mice, PM-FolamiR-34a: *n* = 5 mice, FM-FolamiR-34a: *n* = 6 mice). Arrows represent treatment time (1.5 nmol, intravenous injection, once every 6 days). Error bars: means ± SEM; ^*^p < 0.05, ^**^p < 0.01, ^***^p < 0.001, ^#^*p* < 0.0001; two-way ANOVA with Tukey’s multiple comparisons test. **F** Graph highlighting tumor volume of individual mice treated with the folate-conjugates on day 1 and 21, data normalized to first day of treatment (folate-NC: *n* = 6 mice, PM-FolamiR-34a: *n* = 5 mice, FM-FolamiR-34a: *n* = 6 mice). **G** Body weight measurement throughout the treatment period (error bars: means ± SD). **H** Measurment of IL-6 and TNFα cytokines in FVB.129 immunocompetent mice two hours post injection with PBS, LPS, FM-FolamiR-34a, or PM-FolamiR-34a (error bars: means ± SD, *n* = 4 mice per group, ^***^p < 0.001, ^****^p < 0.0001, one-way ANOVA with Dunnett’s multiple comparison test against LPS).

To advance FM-miR-34a clinically requires a specific, robust, and non-toxic delivery platform. To achieve this, we used a folate-miRNA delivery strategy, which allows for specific delivery of a miRNA to folate receptor (FR) overexpressing tumors without the need for packaging the miRNA within a vehicle [21, 26]. In this case, the naked miRNA is subjected to both serum and cellular nucleases allowing for a full assessment of how the fully modified miRNA would behave in a clinical setting. First, to validate that the folate-conjugates are robustly and specifically delivered to FR expressing cells, cells were treated with folate conjugated to a near-infrared dye (folate-NIR, see Fig. S6A, B for synthesis scheme and validation). Folate-NIR conjugates interacted specifically with FR expressing MB-231, Hela, KB, and IGROV1 cancer cells, as binding was competed away in the presence of excess folic acid (Fig. S6C). Second, to evaluate efficacy in vivo, PM-miR-34a or FM-miR-34a sense strands were conjugated to folate followed by annealing of the respective antisense strands to generate PM-FolamiR-34a and FM-FolamiR-34a duplexes (Fig. S7). To compare the time-dependent repression of targets using the folate-conjugates, mice bearing MB-231-miR-34a sensor cells were injected with a single 1.5 nmol dose of PM-FolamiR-34a, FM-FolamiR-34a, or folate-NC (Fol-NC, folate conjugated to a non-targeting RNA). While the signal in PM-FolamiR-34a treated mice dropped 24–48 h following treatment, the reduction was modest and returned to basal level rapidly. Conversely, the reporter signal in animals treated with a single dose of FM-FolamiR-34a, was robustly downregulated 24 h after systemic injection and remained repressed for at least an additional 96 h (Fig. 6A, B, and Fig. S8A).

While the reporter data are encouraging, the effects on endogenous targets was even more remarkable. Following the 120 h live-animal imaging timepoint, tumors were harvested and endogenous targets of miR-34a were evaluated. Five days following the single 1.5 nmol dose of FM-FolamiR-34a, target genes were robustly downregulated (Fig. 6C, Fig. S8B). Both MET and AXL were undetectable in all three tumors, while CD44 levels were significantly reduced, confirming the ability of FM-miR-34a to effectively silence its biological targets in vivo. The robust and sustained targeting was likely a contribution of increased thermostability between the target and FM-miR-34a, coupled with increased stability of FM-miR-34a, which is highlighted by the >1.5-fold increase in miR-34a copy number in tumors collected from FM-FolamiR-34a treated mice relative to mice treated with a similar dose of PM-FolamiR-34a (Fig. 6D, Fig. S8C).

Based on the outstanding sustained targeting, a multi-dose efficacy study was conducted in MB-231 tumor-bearing mice. In this study, 1.5 nmol of the respective folate-conjugate was administered via tail vein every six days. The rational for this specific dose and frequency of dosing was based on the single-dose experiment which resulted in significant target gene repression for at least five days post-injection (see Fig. 6C). While PM-FolamiR-34a administration led to a delay in tumor growth, the inhibitory effect of FM-FolamiR-34a on tumor growth was more robust, resulting in a clear tumor-static effect (Fig. 6E, F). It is also worth mentioning that tumors in two of the mice administered FM-FolamiR-34a shrunk to ~45–75% of the initial volume by the end of the 21-day dosing period, with one complete cure (Fig. S8D). Importantly, no significant changes were observed in the body weight throughout the study suggesting the safety of FM-FolamiR-34a (Fig. 6G). As a preliminary evaluation of the potential immune response, FM-FolamiR-34a or PM-FolamiR-34a were injected into the tail vein of immunocompetent FVB.129 mice followed by quantification of IL-6 and TNF-α levels in the serum two hours post-injection. Neither FM-FolamiR-34a nor PM-FolamiR-34a caused a significant increase in cytokine levels above the negative control, while the positive control, LPS, caused a marked increase (Fig. 6H). Collectively, these results indicate that FM-FolamiR-34a induces stronger and prolonged silencing of both synthetic and biological targets of miR-34a resulting in a significant delay in tumor growth in comparison to PM-FolamiR-34a in vivo.

## Discussion

miRNA-based therapeutics have emerged as potential therapeutic tools for treating various diseases due to their unique ability to modulate the expression of multiple genes. However, advancing therapeutic miRNAs for clinical use requires optimizations to overcome many of the challenges in the miRNA field, including delivery-associated toxicity and rapid miRNA degradation [1–3]. To overcome these issues, we developed and applied a full chemical modification pattern to the miRNA, miR-34a (FM-miR-34a) and directly conjugated FM-miR-34a to the small molecule folate to achieve specific delivery while retaining stability of the miRNA. Several of the chemical modifications used here have been used extensively for stabilizing siRNAs and ASOs, including 2′-O-methyl and 2′-fluoro ribose bases, and phosphorothioate linkages [5, 30–32]. These modifications not only provide stability against nucleases but also enhance the silencing activity, both culminating in reduced therapeutic dosing. However, only a few studies evaluated the effect of chemical modification on miRNA duplex activity, with none showing efficacy in an in vivo setting [33], a setting where the miRNA would be subjected to several barriers such as exposure to nucleases, poor miRNA uptake, and a complex tumor microenvironment [34–36].

In this study, we directly compared the stability and activity of partially and fully modified miR-34a using lipid transfection and folate-mediated miRNA delivery approaches. We demonstrate that the developed full chemical modifications pattern, when applied to miR-34a, extends its stability in comparison to unmodified or partially modified miR-34a. The global gene regulation analysis also demonstrated that the chemical modification does not hinder, but rather enhances, the ability of miR-34a to downregulate its known/predicted targets. While we observed some initial correlations between target gene repression and phenotypic response, future studies will need to be carried out to verify these early results. For example, in MB-231 breast cancer cells grown in culture and in vivo, FM-miR-34a induced stronger silencing of MET and CD44 in comparison to PM-miR-34a. Consistent with these results, migration and in vivo tumor growth of MB-231 cells transfected with FM-miR-34a was significantly reduced. In prostate cancer cells (LNCaP), FM-miR-34a induced a similar downregulation of AR protein expression and a comparable inhibition of invasion as cells transfected with PM-miR-34a. Whether significant targets contributing to these phenotypes are truly more efficiently downregulated by FM-miR-34a in breast cancer relative to prostate cancer is a future endeavor worth exploring. Clearly, the difference in the magnitude of silencing of MET, CD44, and AR by FM-miR-34a suggests that the modifications likely affect target genes differently. This was further confirmed in our bioinformatics analysis indicating robust downregulation of miR-34a targets by FM-miR-34a in comparison to PM-miR-34a. It is anticipated that varying the modification pattern would alter the effects and targeting of miR-34a, future work that could lead to an even more pronounced effect, or perhaps a tumor-type or pathway-specific response. Indeed, in addition to miR-34a targets, a modest increase in immune-related gene alterations was observed following transfection with FM-miR-34a (Table S3 and Bioinformatics supplementary data). Whether these changes enhance or reduce efficacy in an immunocompetent model would need to be addressed in future studies [21].

Importantly, using a more clinically relevant approach for miRNA delivery (FM-FolamiR-34a), we evaluated the effect of chemical modifications on miR-34a activity in vivo. Based on previous miRNA therapeutic studies, all of which used partially modified miR-34a, full chemical modification of miR-34a results in a more robust response that is sustained, using only 1.5 nmol. Notably, five days following systemic administration of a single dose of FM-FolamiR-34a, biological targets (MET, CD44 and AXL) were silenced to levels commonly observed following single-gene siRNA targeting. There are two main reasons for the enhanced targeting over PM-miR-34. Firstly, the modifications provide enhanced stability, which we verified following quantification of miR-34a in the tumors. Secondly, the 2′-O-methyl modifications increase the T_m_ of the miRNA, which likely facilitate enhanced target gene engagement [37].

One of the most significant bottlenecks with using a ligand-mediated delivery approach is sequestration of the delivered RNA inside of endosomes, away from the cytoplasm where the miRNA is bioactive. Enhancing miRNA stability which prolongs miRNA-mediated targeting could ultimately reduce the need for endosomal escape agents. This was seen in the case of fully modified siRNA conjugated to *N*-acetylgalactosamine (GalNAc) ligand (GalNAc-siRNA) [38]. The in vivo activity of GalNAc-siRNAs is durable, in part due to enhanced stability and slow release from the acidic intracellular compartments [38]. However, future studies are still needed to follow the fate of modified miRNAs within the cells and compare their activity in the presence or absence of endosomal escape agents. Additionally, the pattern of modifications and impact of full chemical modifications of other tumor suppressive miRNAs needs to be evaluated to identify if the enhanced efficacy is sequence/pattern dependent. Another critical aspect that needs to be considered before bringing miRNA therapeutics back into the clinic is the potential immunogenicity. Consistent with previous studies that use chemical modifications for abrogating siRNA-driven immune stimulation [39–41], neither FM-FolamiR-34a nor PM-FolamiR-34a increased early cytokine levels above the negative control level. Despite that, the safety and efficacy of FM-FolamiR-34a will need to be determined in immunocompetent mice and likely larger mammals before clinical advancement.

Nonetheless the work here clearly indicates that full chemical modification of the miR-34a duplex using 2′-O-methyl and 2′-fluoro ribose bases and phosphorothioate linkages enhances both stability and activity of the miRNA. The combination of specific ligand delivery vehicles with chemically modified miRNAs could be beneficial for reducing the effective dose, and for avoiding toxic side effects resulting from non-specific uptake or higher miRNA doses. And, while these stabilizing modifications are useful to withstand serum nucleases, which is needed when delivery is by way of a ligand, the modifications are also essential for increasing intracellular stability. Thus, one could imagine encapsulating FM-miR-34a in larger lipid nanoparticles, which could increase circulation half-life, while still affording protection intracellularly.

## Materials and methods

### Study design

This study was designed to test whether full chemical modification of miR-34a has enhanced stability in comparison to a partially modified version, while retaining its targeting and efficacy in cells in culture. The study further assessed the in vivo therapeutic potential of fully modified miR-34a (FM-miR-34a) when conjugated to a clinically relevant targeting ligand, folate. For in vivo studies, a priori power analysis was used to estimate sample size requiring a statistical significance of 0.05, α < 0.5, and 80% power. On the basis of the power calculation, the suggested number of animals to include in each treatment group was six. One of the animals treated with PM-FolamiR-34a died due to complications unrelated to tumor growth prior to treatment, and thus was removed from the study. Based on the strong and significant response observed using the remaining five mice, no additional mice were treated. For all in vivo work, tumor burden was calculated by caliper measurement and animals were randomized before treatment such that tumor burden and error in each group were equivalent. None of the researchers were blinded to any of the studies conducted. For all treatment groups, the variance between the groups was similar. For all other in vitro studies, sample sizes were based on previous work done in the laboratory.

### Cell culture

MDA-MB-231 (hereafter referred to as MB-231) and LNCaP cells were obtained from ATCC. MB-231 cells selected for high folate receptor expression were a kind gift from Dr. Philip Low (Purdue University). MB-231-miR-34a reporter cells were generated previously [21]. All of the MB-231 strains were cultured in RPMI 1640 medium (no folic acid, Life Technologies), while LNCaP cells (CRL-1740™, ATCC) were cultured in RPMI-1640 medium (30–2001™, ATCC). Both medias were supplemented with 10% fetal bovine serum (FBS; Sigma), penicillin (100 U/mL), and streptomycin (100 mg/mL) (HyClone, GE Healthcare Life Sciences). Cells were monitored monthly for lack of mycoplasma using the MycoAlert Mycoplasma Detection Kit (Lonza). MB-231 cells overexpressing the folate receptor and MB-231-miR-34a sensor cells were authenticated by ATCC using short tandem repeat profiling.

### Preparation of miRNA duplexes and serum stability assay

Unmodified-, partially modified-, and fully modified-miR-34a duplexes were prepared by annealing corresponding sense and antisense strands at an equal molar ratio in the presence of annealing buffer [10 mM Tris buffer, pH 7 (Sigma), 1 mM EDTA (Sigma), 50 mM NaCl (Sigma)] followed by incubation at 95 °C for 5 min, and slow cooling to room temperature. Annealed oligos were then used for cell transfection or otherwise stored at −80 °C. To prepare folate-miRNA conjugates (FolamiRs), the azide-containing sense strand was mixed with folate-dbco (see Fig. S7A, B for synthesis scheme and validation) at a 1:10 molar ratio (sense strand: folate-dbco) and was incubated at 23 °C for 10 h with shaking. The next day, folate-miRNA conjugates were purified using Oligo Clean & Concentrator (Zymo Research) followed by annealing of the antisense strand at an equal molar ratio in the presence of annealing buffer as mentioned above. To assess the stability in serum, miR-34a duplexes (50 pmol) were incubated in 50% FBS (Sigma) at 37 °C for the indicated time points. At each time point, RNA samples were mixed with RNA loading dye and stored at −20 °C. After the last time point, samples were analyzed on a 15% polyacrylamide gel in Glycerol Tolerant Gel Buffer (GTB buffer) followed by staining RNA using Gel Red Nucleic Acid Gel Stain (Thermo Fisher Scientific, Biotium 41003). The sequences of the oligos can be found in Table 1.

**Table 1:** Chemical modification patterns and sequences of miR-34a and the negative controls.

Oligo name	Sequence and modifications
siLuc2 sense strand (PM-NC)	/5AzideN/mGmArAmGrUrGmCrUmCrGmUrCmCrUmCrGmUCCrUrU
siLuc2 antisense strand (PM-NC)	/5Phos/rGrGrArCrGrArGrGrArCrGrArGrCrArCrUmUmCrUrU
miR-34a sense strand (PM)	/5AzideN/mCmArAmCrCmArGmCrUmArAmGrAmCrAmCrUmGrCC
miR-34a antisense strand (PM)	/5Phos/rUrGrGrCrArGrUrGrUrCrUrUrArGrCrUrGrGrUmUmGrU
miR-34a sense strand (FM)	/52FG/*mC*/i2FU/mA/i2FA/mG/i2FA/mC/i2FA/mC/i2FU/mG/i2FC/*mC*/i2FA//3AzideN/
miR-34a antisense strand (FM)	5Phos/mU*/i2FG/*mG/i2FC/mA/i2FG/mU/i2FG/mU/i2FC/mU/i2FU/mA/i2FG/mC/i2FU/mG/i2FG/mU/i2FU/mG*/32FU/
siLuc+ sense strand (FM-NC)	/52FC/*mG*/i2FC/mU/i2FG/mA/i2FG/mU/i2FA/mC/i2FU/mU/i2FC/*mG*/i2FA//3AzideN/
siLuc+ antisense strand (FM-NC)	/5Phos/mU*/i2FC/*mG/i2FA/mA/i2FG/mU/i2FA/mC/i2FU/mC/i2FA/mG/i2FC/mG/i2FU/mA/i2FA/*mG

*PM* Partially modified, *FM* Fully modified, siluc2 and siluc + : Anti-luciferase siRNAs used as a negative control (NC) in the experiments, *miR* miRNA, *m* 2’-O-Methyl, *F* 2’-Fluoro, *r* ribonucleotide, *i* internal, “*” phosphorothioate bond, *Phos* 5’ Phosphate.

### In vitro Renilla Luciferase assay

MB-231 reporter cells were transfected with a negative control (NC) RNA, PM-miR-34a, or FM-miR-34a at the indicated concentrations using Lipofectamine RNAiMAX (Life Technologies). At each time point, Renilla-Glo Luciferase assay (Promega) was performed as per manufacture instructions. In brief, Renilla-Glo Luciferase substrate was mixed with Renilla-Glo buffer at 1:1000 dilution followed by addition into each well. After shaking the plates at room temperature for 10 min, Renilla luciferase signal was measured using a GloMax plate reader (Promega). In the case of Renilla luciferase assay following Ago2 knockdown, MB-231-miR-34a sensor cells were seeded in individual wells of a 96-well plate. The following day, cells were co-transfected with 50 nM siRNA against Ago2 (GeneSolution GS27161; QIAGEN) or a control siRNA (4390846; Thermo Fisher Scientific) along with 10 nM NC, PM-miR-34a, FM-miR-34a duplexes, or miR-34a mimic (MC11030; Ambion) using Lipofectamine RNAiMAX (Life Technologies). Renilla luciferase assay was performed as described above 48 h post-transfection.

### Protein analysis using Western blot

MB-231 or LNCaP cells (1 × 10^5^) were seeded in individual wells of a 24-well plate (coated with poly-D-lysine in case of LNCaP) followed by transfection with 50 nM of PM-miR-34a, FM-miR-34a, or siLuc2 (negative control) using Lipofectamine RNAiMAX (Life Technologies). To quantify the protein expression of miR-34a targets following Ago2 knockdown, MB-231 or LNCaP cells were seeded in individual wells of a 24 well plate followed by co-transfection with 50 nM siRNA against Ago2 (GeneSolution GS27161; QIAGEN) or a control siRNA (4390846; Thermo Fisher Scientific), along with 50 nM NC, PM-miR-34a, FM-miR-34a duplexes, or miR-34a mimic (MC11030; Ambion) using Lipofectamine RNAiMAX (Life Technologies). At each indicated time point, cells were lysed using RIPA buffer [Tris-HCl (pH 8.0, 50 mM), N P-40 (1%), Sodium chloride (150 mM), Sodium deoxycholate (0.5%), SDS (0.1%), ddH2O (up to 100 mL)] in the presence of 1X protease inhibitor cocktail (PIA32955, Thermo Fisher Scientific). Protein concentration was measured using the Pierce BCA Protein Assay kit. Protein lysate (50 μg) was resolved on 12% TGX gels (Bio-Rad) and transferred to polyvinylidene difluoride (PVDF) membranes. After membrane blocking in LI-COR buffer for 1 h at room temperature, the membrane was incubated overnight in the indicated primary antibody at 4 °C. Following incubation with the corresponding secondary antibody, blots were scanned using Li-Cor Odyssey CLX (Li-Cor). Antibodies used: rabbit Androgen receptor (D6F11) XP (5153, Cell Signaling), rabbit MET (D1C2) XP (8198, Cell Signaling), mouse CD44 (156-3C11) (3570, Cell Signaling), mouse β-ACTIN (3700, Cell Signaling), rabbit AXL (C89E7) (8661, Cell Signaling), rabbit GAPDH (14C10) (2118, Cell Signaling), and rabbit anti-Ago2, clone 11A9 (MABE253; Millipore). All the antibodies were used at 1:1000 dilution except the following: anti-Ago, clone 2A8 (MABE56; Millipore), and rabbit AXL (C89E7) (8661, Cell Signaling) were used at 1:500 dilution.

### mRNA quantification using qRT-PCR

MB-231 cells (1 × 10^5^) were seeded in individual wells of a 24 well plate. The next day, cells were transfected with 50 nM PM-miR-34a, FM-miR-34a, or siLuc2 (negative control) using Lipofectamine RNAiMAX (Life Technologies). Forty-eight hours later, total RNA was isolated using the miRneasy Kit (217004, Qiagen) according to the manufacturer’s instruction. After removal of genomic DNA using DNase I digestion (79254, Qiagen), RNA integrity was evaluated by resolving on a 1.5% agarose gel. RNA concentration was quantified using a nanodrop. Total RNA (500 ng) was used to generate cDNA using the miScript Reverse Transcriptase kit (218161, Qiagen) using HiFlex buffer per the manufacturer’s instructions. Real-time polymerase chain reaction (qPCR) was performed using the SYBR Green PCR Kit (QIAGEN) with the following primers: Hs_AXL_1_SG, Hs_SIRT1_1_SG, Hs_MET_1_SG, Hs_GAPDH_1_SG, Hs_GNB2L1_2_SG, Hs_TNS4_1_SG, and Hs_ACTB_1_SG (QuantiTect Primer Assay; QIAGEN). Data were then analyzed using the 2^−ΔΔCt^ method [42] and expressed as fold change.

### Cell proliferation assays

The Sulforhodamine B (SRB, Sigma) assay was used to measure cell proliferation as previously reported [43]. In brief, MB-231 or LNCaP cells were seeded onto individual wells of a 96-well plate (coated with poly-D-lysine in case of LNCaP). The next day, cells were transfected with the various miRNA duplexes (50 nM in case of MB-231 and 10 nM in case of LNCaP) using Lipofectamine RNAiMAX (Life Technologies). At the indicated time points, cells were fixed using 10% tricholoroacetic acid in complete media for 1 h at 4 °C. Afterward, cells were stained with 0.04% (wt/vol) SRB in 1% acetic acid for 1 h at 37 °C followed by washing unbound dye five times with 1% acetic acid. Unbuffered Tris base (10 mM) was used to extract protein-bound dye and absorbance at 510 nm, which is a proxy for cell mass, was measured using a GloMax Multi+ spectrophotometer (Promega). For clonogenic assays, transfected MB-231 cells were counted and plated at the density of 250 cells/well in 6 well plate. At the indicated time points, cells were stained using the Differential Quik^®^ staining kit (Polysciences, cat no. 26419-16). Number of colonies were quantified using ImageJ v1.53t (NIH). Images were converted to RGB stack and for the highest contrast stack, threshold was adjusted to “10–140” and analyzing particles by setting size (pixel^2^) to “50-Infinity”. The data was compiled and analyzed using GraphPad Prism v9.4.1 (GraphPad Software, LLC).

### Cell migration and invasion assay

For migration assays, 2 × 10^5^ MB-231 cells were seeded in each well of a 6-well plate. After 24 h, cells were transfected with 5 nM PM-miR-34a, FM-miR-34a, or NC (siLuc2) in 50% complete media using Lipofectamine RNAiMAX (Life Technologies) as per manufacturer instructions. Following 72 h of transfection, the cells were trypsinized, counted and 6 × 10^4^ cells from each treatment were transferred to the apical chamber of 5 µm pore-size transwell plates (07-200-149; Fisher Scientific). Basal media was added to the apical chamber and media containing 20% FBS was added to the basolateral chamber. After 12 h, cells were fixed and stained using Differential Quik^®^ staining kit (26419-16; Polysciences, Inc.) as per manufacturer instructions. For invasion assays, 2 × 10^5^ LNCaP cells were seeded and transfected as mentioned above. Following transfection, 5 × 10^4^ cells were transferred to the apical chamber, coated with 100 µl of 200 µg/ml of Matrigel matrix (08-774-122; Fisher Scientific) at 37 °C for 1 h, of 8 µm pore-size transwell plates (07-200-150; Fisher Scientific).

To image migration or invasion chambers, cells were removed from the apical side of the porous membrane using cotton tip applicators and the insert was placed on a glass slide. Four fields were randomly selected, imaged using Olympus IX73 microscope at 10X magnification. Number of cells were quantified using ImageJ v1.53t (NIH). Images were converted to RGB stack and for the highest contrast stack, threshold was adjusted to “0–90” and analyzing particles by setting size (pixel^2^) to “50-Infinity”. The data was compiled and analyzed using GraphPad Prism v9.4.1 (GraphPad Software, LLC).

### RNA sequencing

MB-231 cells (2 × 10^5^) were seeded into individual wells of a 6 well plate. The next day, cells were transfected with the various miRNA duplexes (50 nM) using Lipofectamine RNAiMAX (Life Technologies). RNA was extracted from the cells after 48 h using mirVana™ RNA Isolation Kit (Thermo Fisher, AM1560) including removal of genomic DNA using Dnase I digestion (79254, Qiagen). Quantification and purity of samples were determined by nanodrop, and samples integrity was confirmed using Agilent bioanalyzer (Agilent Technology, California USA). RNA sequencing libraries were prepared using poly A enrichment method using NEBNext® Ultra™ II RNA Library Prep Kit for Illumina® to remove ribosomal RNA. The libraries were then checked with Qubit and real-time PCR for quantification, and bioanalyzer for size distribution detection. RNA sequencing was performed using NovaSeq 6000 platform with a paired end 150 base pair strategy.

### Bioinformatics analysis

The raw reads were trimmed and aligned to GRCh38 (Ensembl release 104). DESeq2 (v1.36.0) was used to normalize the read count and determine differentially expressed genes [44]. The p-value cutoff for statistically significant genes was 0.05 and no cutoff was used for log_2_FC. Volcano plots were plotted using *EnhancedVolcano* package (v1.14.0) in R. Area-proportional Venn diagrams were adapted from DeepVenn tool [45]. Heatmaps were generated using *pheatmap* package (v1.0.12) in R with distance measure set to “Euclidean” and clustering method set to “ward.D2”. Gene set enrichment and miRNA target enrichment analysis was performed using *gprofiler2* package (v0.2.1) in R with statistical significance computed using g:SCS algorithm and set to 0.05 [46]. Terms with p-adj (corrected *p*-value) <0.05 were selected for further analysis. The data for miRNA target enrichment analysis was exported and visualized using *ggplot2* package (v3.3.6) in R. For miR-34a known/predicted target analysis, targets were exported from miRDB database [47] and overlapped with genes downregulated in PM-miR-34a vs NC and FM-miR-34a vs NC comparisons. GraphPad Prism v9.5.0 (GraphPad Software, LLC) was used to visualize the results. For analysis of immune-related genes, Human Immunology v2 panel (XT-CSO-HIM2–12, Nanostring Technologies) was used, which consists of 594 genes (579 well-annotated immune response genes and 15 housekeeping genes). All R analysis was performed using statistically significant genes (*p* < 0.05) or gene ontology terms (p-adj < 0.05) and was conducted in RStudio environment (v2022.12.0 + 353).

### RNA immunoprecipitation assay

MB-231 cells (3 × 10^6^) were seeded in 10 cm plates. The next day, two plates of cells were transfected with 10 nM of each of miR-34a mimic (Ambion), PM-miR-34a, FM-miR-34a, or siLuc2 (negative control) duplexes using Lipofectamine RNAiMAX (Life Technologies). The transfection media was replaced with complete media four hours post-transfection. Twenty-four hours later, the culture media was discarded, and cells were washed twice with ice-cold PBS before cross linking at 400 mJ/cm^2^ and then again at 200 mJ/cm^2^ using a UV cross-linker (XL-1000; SpectroLinker). Cell lysis buffer (1 x PBS, 1% vol/vol NP40, 0.5% wt/vol sodium deoxycholate, and 0.1% wt/vol SDS) was added to each plate in the presence of 1x protease inhibitor cocktail (PIA32955, Thermo Fisher Scientific) and RNase inhibitor (AM2696; Invitrogen) for 30 min with shaking at 4 °C. After shaking, the cells were scraped into 1.5 ml microcentrifuge tubes and DNase I (79254, Qiagen) was added to remove genomic DNA. The resulting cell lysates were centrifuged at 16,000 x *g* for 20 min at 4 °C and the supernatants were pre-cleared by incubating with 20μl Dynabead Protein A beads (Life Technologies). Pre-cleared cell lysates were incubated with 2A8 anti-Ago (MABE56; Millipore) or normal mouse IgG (12–371; Millipore) overnight at 4 °C. Afterward, Dynabead Protein A beads that were linked to the bridging antibody, Rabbit anti-mouse IgG Fcγ (NC9549822; Fisher Scientific), were added to each sample. Samples were incubated for 2 h at 4 °C followed by washing and resuspending the beads as previously described [48]. RNA was extracted using Qiazol reagent (Qiagen) followed by ethanol precipitation. The miRscript II RT kit (Qiagen) was used to generate cDNA using the HiSpec buffer followed by quantitative reverse transcription polymerase chain reaction (qRT-PCR) using the SYBR Green PCR Kit (Qiagen). The following primers were used: mir-34a-5p (miScript primer assay; Qiagen) and RNU6B (non-target RNA, miScript primer assay; Qiagen). Data were then analyzed using the 2^−ΔΔCt^ method and expressed as fold change.

### Tumor implantation and in vivo experiments

To evaluate the effect of FM-miR-34a on tumor growth, MB-231 cells were transfected with 50 nM PM-miR-34a, FM-miR-34a, or NC (siLuc2) using Lipofectamine RNAiMAX (Life Technologies) in 10 cm plates. Twenty-four hours later, cells were trypsinized, washed with 1x PBS, and mixed with Matrigel (Corning) at a 1:1 dilution. Cells (5 × 10^6^) were subcutaneously injected into the left and right flanks of 8–10 week-old female (NU/J, Foxn1^nu^, strain #: 002019, Jackson Lab) mice. A vernier caliper was used to measure tumor volume at the indicated time points which was calculated using the following formula: tumor volume: length × width^2^/2.

For the single-dose study using FolamiRs, MB-231-miR-34a sensor cells (7 × 10^6^) were injected into the flank of 10–12 week-old female (NU/J, Foxn1^nu^, strain #: 002019, Jackson Lab) mice, which were maintained on a folate-deficient diet (TD.95247, Envigo) for 1 week prior to treatment and during the course of the experiment. When the tumor volume reached ~200 mm^3^, mice were treated with a single dose of folate-NC (siLuc2), PM-FolamiR-34a, or FM-FolamiR-34a (1.5 nmol) via tail vein injection. Luminescent signals were captured prior to treatment and over the course of 120 h using Coelenterazine h Bioluminescent Substrate (PerkinElmer), which was administered intraperitoneally per the manufacturer’s instructions. Whole animal imaging was performed using Spectral AMI (Spectral Instruments). For extraction of protein and RNA from the tumor samples, individual tumors were harvested and stored in RNA later (Life Technologies) at −80 °C until processing. Tumor tissues (50 mg) were disrupted by grinding using liquid nitrogen in a cold mortar. The powder from each tumor sample was transferred into an Eppendorf tube followed by addition of RIPA buffer [Tris-HCl (pH 8.0, 50 mM), NP-40 (1%), Sodium chloride (150 mM), Sodium deoxycholate (0.5%), SDS (0.1%), ddH2O (up to 100 mL)] in the presence of 1X protease inhibitor cocktail (PIA32955, Thermo Fisher Scientific). Following centrifugation, an equal amount of protein lysate (50 µg) was resolved on TGX gels (Bio-Rad) followed by analysis of protein by immuno-detection. Total RNA was extracted from the tumor samples using mirVana™ RNA Isolation Kit (Invitrogen™, AM1560). cDNA was prepared using miScript II RT Kit (Qiagen) with the supplied HiSpec Buffer using 1 μg of total RNA. A standard curve (1 × 10^3^ copies to 1 × 10^8^ copies) was generated using the miR-34a mimic (Life Technologies). qRT-PCR reaction (At least 3 technical repeats per biological replicate) was performed using the miScript SYBR Green PCR Kit (Qiagen) and miRNA primer assays (Qiagen) in a QuantStudio 6 Flex Real-time PCR machine (Life Technologies).

To determine the efficacy of FM-FolamiR-34a conjugates on tumor growth, MB-231 cells (5 × 10^6^) were injected into the flank of 8–10 week-old female (NU/J, Foxn1^nu^, strain #: 002019, Jackson Lab) mice, which were maintained on a folate-deficient diet (TD.95247, Envigo). When the tumor volume reached ~150 mm^3^, mice were treated with 1.5 nmol folate-NC (siLuc2), PM-FolamiR-34a, or FM-FolamiR-34a via tail vein once every 6 days. Body weight was recorded, and the tumor volume of each mouse was measured every 3 days using a vernier caliper and was calculated using the following formula: tumor volume: (length × width^2^)/2. All protocols were approved by the Purdue Animal Care and Use Committee and guidelines set forth by the National Institutes of Health (NIH) guidelines for animal use were followed.

### Quantification of serum cytokines

To evaluate potential immune response in vivo, immune-competent mice (FVB.129 background) were injected with Lipopolysaccharide (LPS, 0.63 mg/kg, intraperitoneally), PBS, FM-FolamiR-34a, or PM-FolamiR-34a (1.5 nmol, *n* = 4, tail vein). Two hours after injections, mice were euthanized, and whole blood was collected. Whole blood was incubated at room temperature for 1 h, at which time serum was collected by centrifugation at 2,000 x *g* for 10 min in a refrigerated centrifuge followed by storage at −80 °C until cytokine analysis. Interleukin-6 (IL-6) and tumor necrosis factor alpha (TNF-α) levels were measured from the serum samples using ELISA Max Deluxe Kit (Biolegend), according to the manufacturer’s instructions.

### Statistical analysis

Statistical analysis was performed using Prism statistical package (GraphPad Software, version 9). The two-tailed Student’s *t-*test was used to determine the statistical difference between two groups. One-way or two-way ANOVA was used to compare the differences between multiple groups and multiple comparisons were corrected using Dunnett’s post hoc test or Tucky’s post hoc test. Data are presented as means ± SD or means ± SEM as specified in the figure legends. Statistically significant *p*-values are as indicated in the corresponding figure legends.

### Supplementary information

General Supplemental

Supplemental Bioinformatics

Supplemental Table 1

Supplemental Table 2

Supplemental Table 3

## Data Availability

RNA-seq data were deposited in the Gene Expression Omnibus database under accession #GSE237836.

## Code availability

The analysis code can be found on GitHub: https://github.com/isohal/RNAseq-analysis.
