# Supplementary material for: A first-in-class fully modified version of miR-34a with outstanding stability, activity, and anti-tumor efficacy
Source: Oncogene. 2023 Sep 5;42(40):2985–99. doi: 10.1038/s41388-023-02801-8 (PMC10541324; doi:10.1038/s41388-023-02801-8)
Supplement: Supplementary file 1 — General Supplemental [file 41388_2023_2801_MOESM1_ESM.docx]

**A first-in-class fully modified version of miR-34a with outstanding stability, activity, and anti-tumor efficacy**

Ahmed M. Abdelaal^1^, Ikjot S. Sohal^1*^, Shreyas Iyer^1^, Kasireddy Sudarshan^2^, Harish Kothandaraman^3^, Nadia A. Lanman^3,4^, Philip S. Low^2,3^, and Andrea L. Kasinski^1,3,*^

^1^Department of Biological Sciences, ^2^Department of Chemistry, ^3^Purdue Institute for Cancer Research, ^4^Department of Comparative Pathobiology, Purdue University West Lafayette, Indiana, 47907, USA

**The file includes:**

**Materials and Methods**

**fig. S1:** Comparison of cellular activity of partially and fully modified miR-34a duplexes.

**fig. S2:** Global gene regulation comparison between PM-miR-34a and FM-miR-34a.

**fig. S3.** Comparative analysis of targeting of miR-34a targets by PM-miR-34a and FM-miR-34a.

**fig. S4:** Evaluation of FM-miR-34a effect on proliferation of BEAS-2B cells.

**fig. S5:** Verification of Ago pull down or knockdown.

**fig. S6:** OTL-38 (Folate-NIR) synthesis, LC-MS spectral analysis and binding to folate receptor (FR) expressing cells.

**fig. S7:** Folate-DBCO ligand synthesis, LC-MS spectral analysis, and validation of PM-FolamiR-34a and FM-FolamiR-34a conjugation

**fig. S8:** Effect of FM-FolamiR-34a and PM-FolamiR-34a on MB-231-miR-34a sensor cells and tumor growth *in vivo*.

**Materials and Methods**

All the reactions were carried out in oven dried glassware. Reactions requiring an inert atmosphere were carried out under an argon atmosphere. Amino acids for peptide synthesis were purchased from Chem-Impex International (Chicago, IL). NHS-DBCO was purchased from Broadpharm (San Diego, CA), S0456 dye was purchased from Few Chemicals (Bitterfeld-Wolfen, Germany) and all other chemical reagents were purchased from Sigma-Aldrich (St. Louis, MO). All conjugates were purified by preparative reverse phase (RP)-HPLC (Agilent) and LC/MS analyses were obtained using an Agilent mass spectrometer coupled with a UV diode array detector.

**Folate-DBCO synthesis**

**N^10^-(trifluoroacetyl)pteroic acid synthesis**:

To the vacuum dried pteroic acid (1 g, 3.2 mmol) in a round bottom flask trifluoroacetic anhydride (23.16 mL, 170 mmol) was added dropwise under argon gas. The round bottom flask was covered with aluminum foil and the contents in the flask were stirred for four days under argon gas. The progress of the reaction was monitored by LC-MS. After the complete consumption of pteroic acid, excess trifluoroacetic anhydride was removed by a rotary evaporator. 25 mL of 3% trifluoroacetic acid was added to the flask and the contents were stirred for two days at room temperature. After this time, 20 mL of HPLC-grade water was added to the flask and the contents in the flask were transferred into centrifuge tubes and centrifuged at 3,000 rpm for 20 minutes. The resultant pellet was washed with HPLC grade water (3 times) followed by centrifugation. The supernatant layer was discarded, and the pellet was lyophilized. The resultant yellow solid (N^10^-trifluoroacetylpteroic acid) was stored in an amber vial.

**Folate-EDA (ethylene diamine) synthesis**:

Folate-Dibenzocyclooctane (DBCO) conjugate was synthesized starting from folate ethylenediamine (EDA) conjugate according to the procedure reported by us.^1^ Briefly, Folate-EDA conjugate was synthesized by following the Fmoc-solid phase peptide synthesis procedure. In a solid phase peptide synthesis vessel, ethylenediamine polymer bound resin (200-400 mesh, 0.5 g, 0.9 mmol/g, 1 eq.) was swollen with 10 mL of dichloromethane followed by 10 mL of dimethylformamide for 30 mins each. A solution of Fmoc-Glu-O^t^Bu solution (0.5 g, 1.125 mmol, 2.5 eq), (Benzotriazol-**1**-yloxy) tripyrrolidinophosphonium hexafluoro phosphate (PyBOP, 0.6 g, 1.125 mmol, 2.5. eq.) and *N, N*-Diisopropylethylamine (DIPEA, 0.481 mL, 2.7 mmol, 3 eq.) in DMF (15 mL) was added. Argon was bubbled for 12 h, after this time, the coupling solution was drained, and the resin was washed with DMF (3x10 mL) and *i*-PrOH (3x10 mL). The efficiency of coupling is monitored by pilot resin cleavage. The Fmoc group was removed with a piperidine solution (20% in DMF, 3x 10 ml) and the resin was washed with DMF (3 x 15 mL) and i-PrOH (3x15 mL). The above coupling reaction sequence is repeated with TFA (trifluoro acetyl)-pteroic acid (0.414 g, 0.9 mmol), pyBOP (0.468 g, 0.9 mmol), and DIPEA (160 uL, 0.9 mmol). After 12 h, the coupling solution was drained, and the resin was washed with DMF (3x10 mL) and i-PrOH (3x10 mL). The resin was treated with 50% NH_4_OH in DMF solution (3x15 mL) for two hours each time. After the 50% NH_4_OH treatment, the resin was washed with DMF (3x15 mL) and i-PrOH (3x15 mL). The resin was dried for 30 min. Folate peptide was cleaved from the resin using a cocktail cleavage solution containing 92.5% trifluoroacetic acid, 2.5% water, and 2.5% triisopropylsilane (3x15 mL) and was bubbled for 2 h. The cleavage mixture was collected in a clean round bottom flask and the combined mixture was concentrated under reduced pressure to a smaller volume. The concentrated product was precipitated in diethyl ether. The precipitate was collected by centrifugation, washed with ethyl ether (3x50 mL), and dried under a vacuum. The crude conjugate was purified by RP-HPLC [A = 20 mM ammonium acetate buffer (pH 7.0), B = acetonitrile, solvent gradient: 5% B to 95% B in 60 min] to yield Folate-EDA compound as yellow solid (38 % yield). LC-MS (A = 20 mM ammonium bicarbonate, pH = 7; organic phase B = acetonitrile; method: 0% B to 30% B in 12 minutes) RT = 3.26 min (M+H^+^ = 484.0).

**Folate-DBCO conjugate synthesis**:

NHS-DBCO (0.0091 g, 0.0227 mmol, 1.1 eq.) and DIPEA (2 uL, 0.0309 mmol, 2 eq.) were added under an inert atmosphere to a solution of Folate-EDA (0.010 g, 0.0206 mmol, 1 eq.) in anhydrous DMSO. The reaction mixture continued with stirring at room temp. The progress of the reaction was monitored by LC-MS. After the complete conversion of Folate-EDA, the crude reaction mixture was purified by RP-HPLC (mobile phase A = 20mM ammonium acetate, pH = 7; organic phase B = acetonitrile; method: 0% B to 50% B in 35 minutes at 13 mL/min) and furnished Folate-DBCO at 82% yield. LC-MS (A = 20 mM ammonium bicarbonate, pH = 7; organic phase B = acetonitrile; method: 0% B to 100% B in 7 minutes) RT = 3.2 min (M+H^+^) = 771.3

**Synthesis of OTL-38**

To the vacuum dried (N^10^-trifluoroacetylpteroic acid) (1 g, 2.5 mmol, 1 eq.) in a round bottom flask in anhydrous DMF (25 mL), HATU (1.14 g, 2.5 mmol, 1.2 eq.) and *O*-*t*-butyl-L-tyrosine t-butyl ester hydrochloride (1.14 g, 2.5 mmol, 1.2 eq.) were added under argon. The contents of the flask were allowed to stir and DIPEA (1.741 mL, 10 mmol, 4 eq.) was added dropwise over a period of 5 minutes. The progress of the reaction was monitored by LC/MS. After 2 h, the contents from the flask were poured slowly into 0.1 N HCl solution (250 mL) to give a pale-yellow precipitate. The obtained precipitate was filtered using a sintered funnel and washed with water (8 × 50 mL) until the pH of the filtrate was between 3 and 4. The wet solid was allowed to dry under a high vacuum for 12 h to obtain titled compound **1** (1.5 g, 88.2%). Analytical UPLC: R_t_ = 3.78 min [solvent gradient: 0% B to 50% B in 5 min].

**Synthesis of Pteroyl-N^10^(TFA)-Tyr(OH)-CO_2_H** (**2**):

The tertiary butyl groups in **1** (1 g) were deprotected using a cocktail cleavage solution containing of 92.5% trifluoroacetic acid, 2.5% water, and 2.5% triisopropylsilane (30 mL) for 2 h. The progress of the reaction was monitored by LC/MS. After the complete consumption of starting material, the contents were added to the tertiary butyl ether (MTBE, 6 × 50 mL) to give a pale-yellow precipitate. The precipitate was filtered using a sintered funnel, washed with MTBE (6 × 50 mL), and dried under vacuum to obtain compound **2**.

**Synthesis of OTL38**:

Compound **2** (0.5 g, 0.875 mmol, 1 eq.) was dissolved in water (20 mL) and the pH of the solution was maintained at 9.5 by adding aqueous 3.75 M NaOH dropwise to result in a pale-yellow solution. The resulting pale-yellow solution was then added dropwise to a solution of S0456-Cl trisodium salt (0.9 g, 0.95 mmol, 0.95 eq.) in water (40 mL) in a round bottom flask. The pH of the resulting solution was maintained at 9.5. The contents of the flask were stirred at 90 ^0^C for 45 mins. The progress of the reaction was monitored by LC/MS. Upon completion of the product formation, the reaction mixture was cooled to room temperature and transferred to acetone (volume) to give a green precipitate. The obtained precipitate was filtered using a sintered funnel and washed with acetone (8 × 50 mL). The green powdery solid was transferred into an amber vial and was dried under a high vacuum. The crude solid was purified using prep-HPLC to obtain pure OTL-38.

## *In vitro* Renilla Luciferase assay

MB-231 reporter cells were transfected with a negative control (NC) RNA, PM-miR-34a, or FM-miR-34a at the indicated concentrations using Lipofectamine RNAiMAX (Life Technologies). At each time point, Renilla-Glo Luciferase assay (Promega) was performed as per manufacture instructions. In brief, Renilla-Glo Luciferase substrate was mixed with Renilla-Glo buffer at 1:1000 dilution followed by addition into each well. After shaking the plates at room temperature for 10 minutes, Renilla luciferase signal was measured using a GloMax plate reader (Promega). In addition, BEAS-2B cells were co-transfected with 50 ng of pmiRGlo-miR-34a sensor and 10 nM PM-miR-34a, FM-miR-34a or the negative controls followed by measuring both Firefly and Renilla signals using Dual-Glo Luciferase assay (Promega) as per manufacture instructions.

**Flow cytometry:**

To evaluate the binding of folate-NIR to folate receptor expressing cells, MB-231, Hela, IGROV1 or KB cells were incubated with 100 nM folate-NIR in the presence or absence of 10 µM folic acid in PBS. Cells were incubated on ice for 1 hour followed by washing with PBS. After resuspending the pellets in PBS, the fluorescence intensity was measured using LSRFortessa flow cytometer (BD Biosciences). Data was analyzed using FlowJo software v10 (Tree Star, Inc, OR, USA).

**Cell proliferation assay and flourscence imaging:**

BEAS-2B cells were seeded onto individual wells of a 96 well plate. The next day, cells were transfected with 10 nM miRNA duplexes using Lipofectamine RNAiMAX (Life Technologies). At the indicated time points, cells were fixed using 10% tricholoroacetic acid in complete media for 1 hour at 4 °C. Afterward, cells were stained with 0.04% (wt/vol) SRB in 1% acetic acid for 1 hour at 37°C followed by washing unbound dye five times with 1% acetic acid. Unbuffered Tris base (10 mM) was used to extract protein-bound dye and absorbance at 510 nm, which is a proxy for cell mass, was measured using a GloMax Multi+ spectrophotometer (Promega). To validate the transfection efficiency, BEAS-2B cells were transfected with 10 nM fluorescently labelled miRNA (miRidian) followed by imaging using Olympus IX73 fluorescence microscope at 10X magnification 24 hrs post transfection.

**
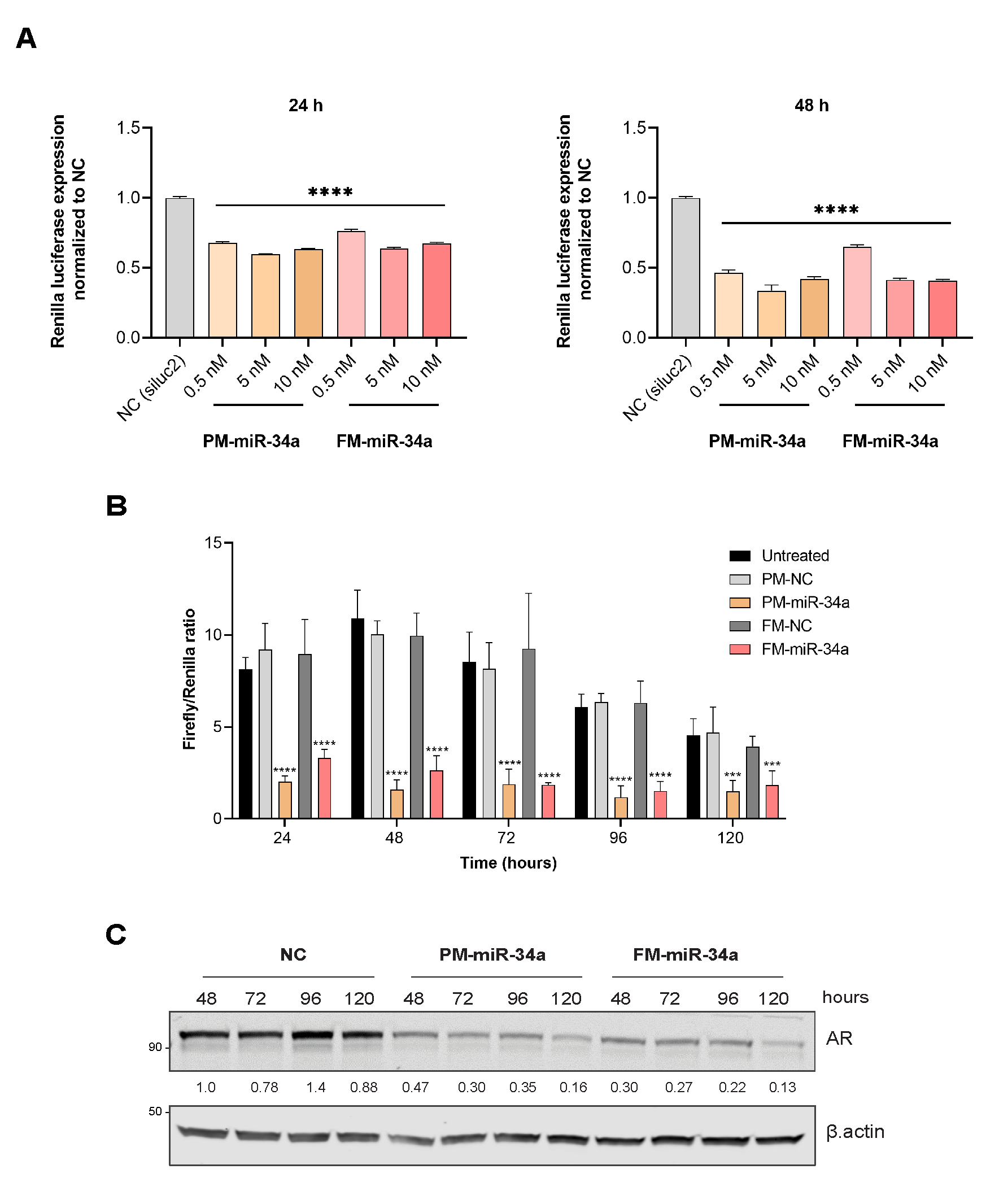
fig. S1. Comparison of cellular activity of partially and fully modified miR-34a duplexes.** **A**, Renilla luciferase expression at 24 h and 48 h post-transfection of MB-231-miR-34a sensor cells with various doses of PM-miR-34a and FM-miR-34a. One representative experiment of n=3 biological replicates is shown, data normalized to NC. **B**, Normalized firefly luciferase signal in BEAS-2B cells following co-transfection with a pmiRGlo-miR-34a sensor and PM-miR-34a, FM-miR-34a, or NC (n=3). **C,** Representative immunoblot image indicating reduction of androgen receptor (AR) expression post-transfection of LNCaP cells with 50 nM FM-miR-34a or PM-miR-34a duplexes at the indicated time points**.** Fold change, normalized to GAPDH and relative to NC (48 hours) is shown below the AR blot. Error bars: means ± SD. ***P < 0.001, ****P < 0.0001), one-way Anova with Dunnett’s multiple comparison test against NC (Siluc2) in A and against untreated in B.


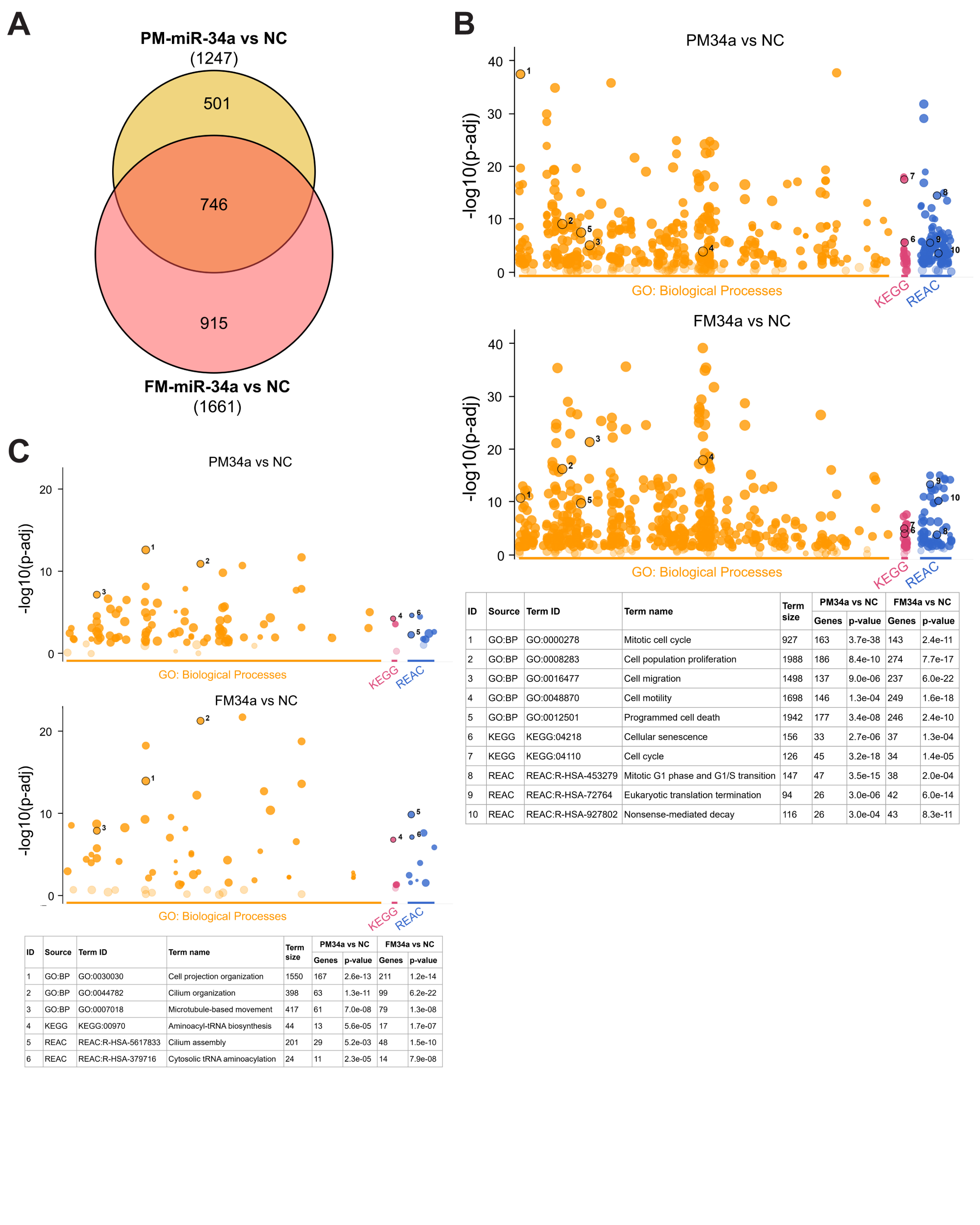


**fig. S2. Global gene regulation comparison between PM-miR-34a and FM-miR-34a transfected cells. A**, Overlap of statistically significant upregulated genes in PM-miR-34a vs NC and FM-miR-34a vs NC comparisons. B, Gene set enrichment analysis of biological processes, KEGG pathways and REACTOME pathways based on genes downregulated following transfection: PM-miR-34a vs. NC and FM-miR-34a vs. NC comparisons. Selected terms are highlighted in the Manhattan plot and their corresponding name, size and adjusted p-values are in the table below. C, Gene set enrichment analysis of biological processes, KEGG pathways and REACTOME pathways based on genes upregulated following transfection: PM-miR-34a vs. NC and FM-miR-34a vs. NC comparisons. Selected terms are highlighted in the Manhattan plot and their corresponding name, size and adjusted p-values are in the table below.


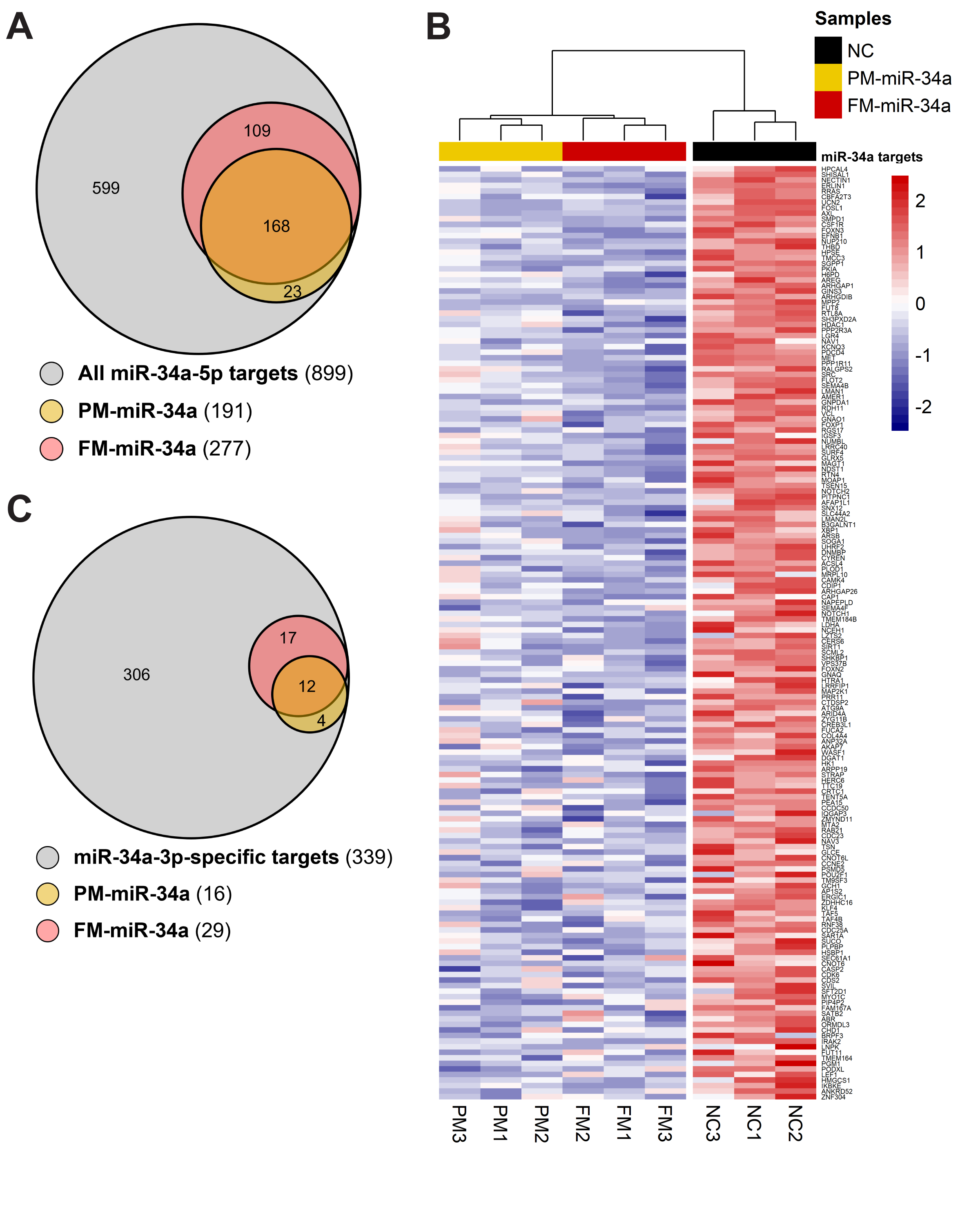


**fig. S3. Comparative analysis of targeting of miR-34a targets by PM-miR-34a and FM-miR-34a.** A, Overlap of all known/predicted miR-34a-5p targets (miRDB) downregulated in PM-miR-34a vs. NC or FM-miR-34a vs. NC comparison. B, Heatmap of miR-34a targets downregulated by PM-miR-34a or FM-miR-34a; columns are clustered based on distance method = “euclidean”, clustering method = “ward.D2”, rows are sorted from lowest-to-highest average fold-change values for FM-miR-34a vs UT comparison. **C**, Overlap of all known/predicted miR-34a-3p (passenger strand) targets (miRDB) downregulated in PM-miR-34a vs. NC or FM-miR-34a vs.NC comparison.


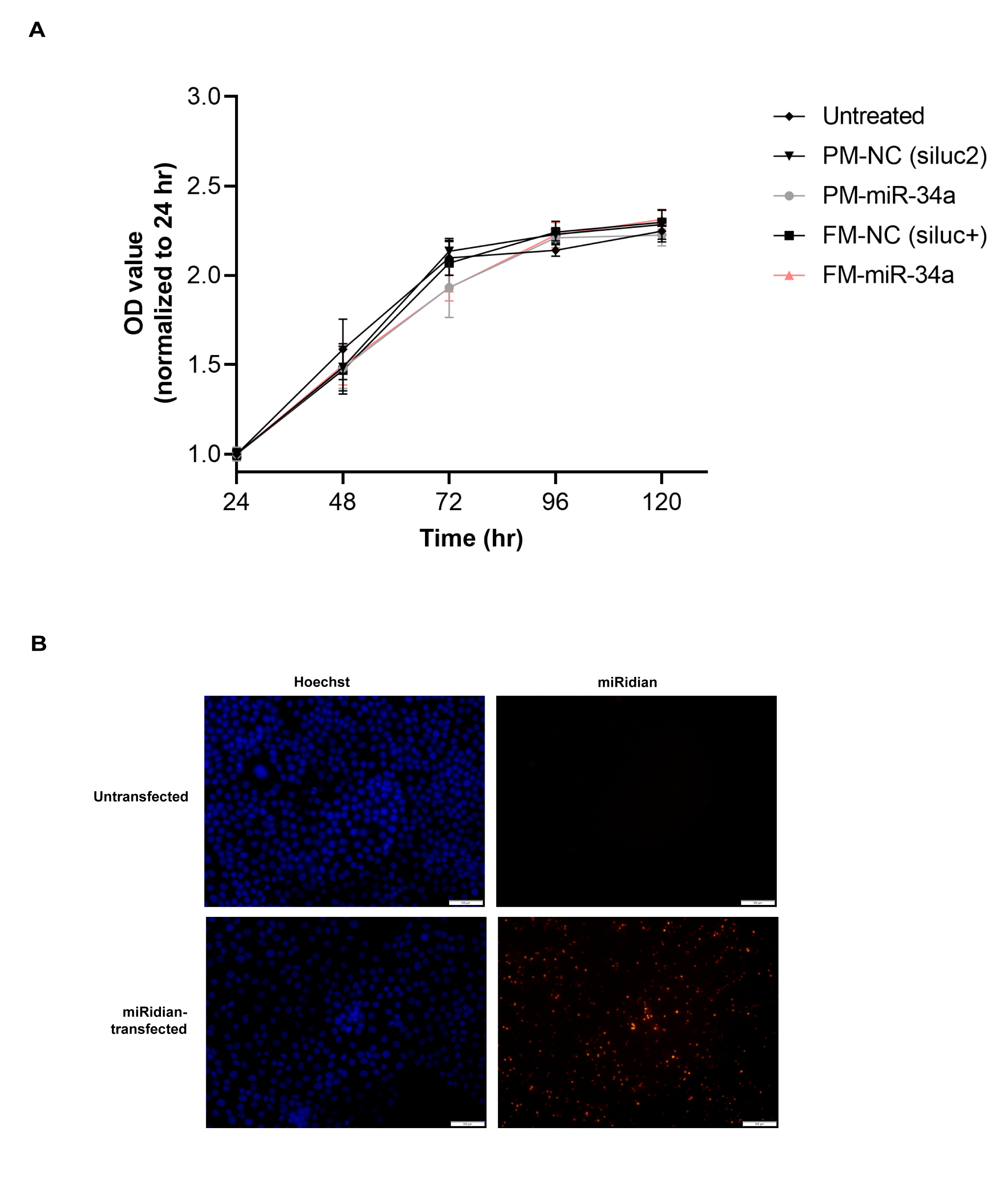
**fig. S4. Evaluation of FM-miR-34a effect on proliferation of BEAS-2B cells. A**, Proliferation of BEAS-2B cells measured by SRB assay post transfection with 10 nM FM-miR-34a or PM-miR-34a at different time points using lipofectamine RNAimax, Data normalized to 24 hours, (error bars: mean ± SD, n= 2 biological replicates with 6 technical replicates per experiment. **B**, BEAS-2B cells were transfected with 10 nM fluorescently labelled miRNA (miRidian) using lipofectamine RNAimax to verify adequate transfection efficiency. Fluorescence microscopic images of miRidian highlight transfection efficiencies of at least 50% (right).


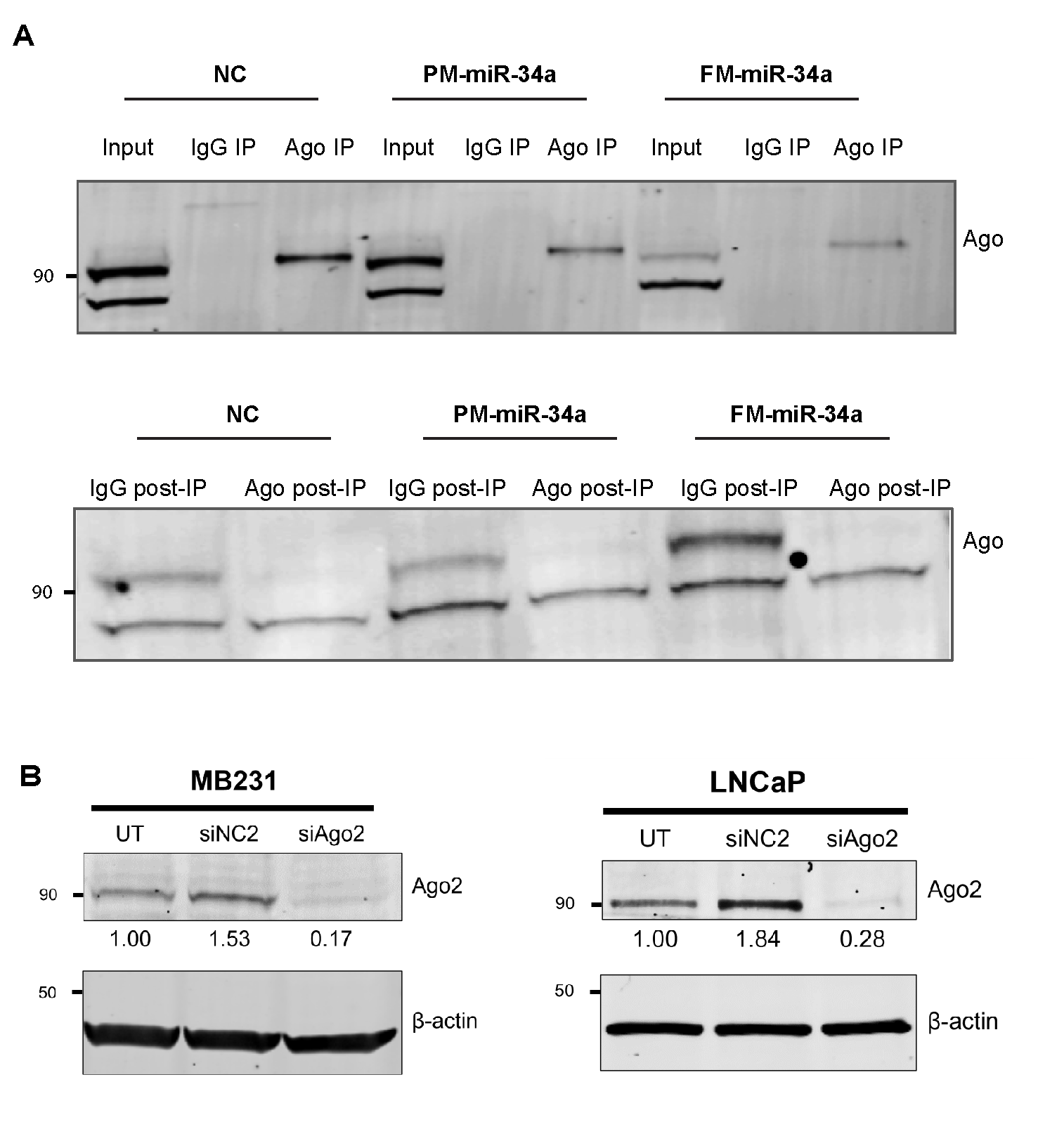


**fig. S5. Verification of Ago pull down or knockdown. A**, Immunoblot images of MB-231 lysate indicating successful pull down of Ago in the Ago-immunoprecipitated fraction (verified before using for RNA immunoprecipitation experiments in Figure 5). The additional lower molecular weight band in the input samples is radixin, which is likely lost during the immunoprecipitation due to the stringent wash conditions as shown in the lower blot of the Ago post-IP samples[1, 2]. **B**, Immunoblot images highlighting successful knockdown of Ago2 following transfection of MB-231 or LNCaP cells with 50 nM siRNA against Ago2 or a control siRNA (siNC2) for 72 hours. Fold change, normalized to β-actin and relative to untreated (UT) is shown below Ago2 blot.


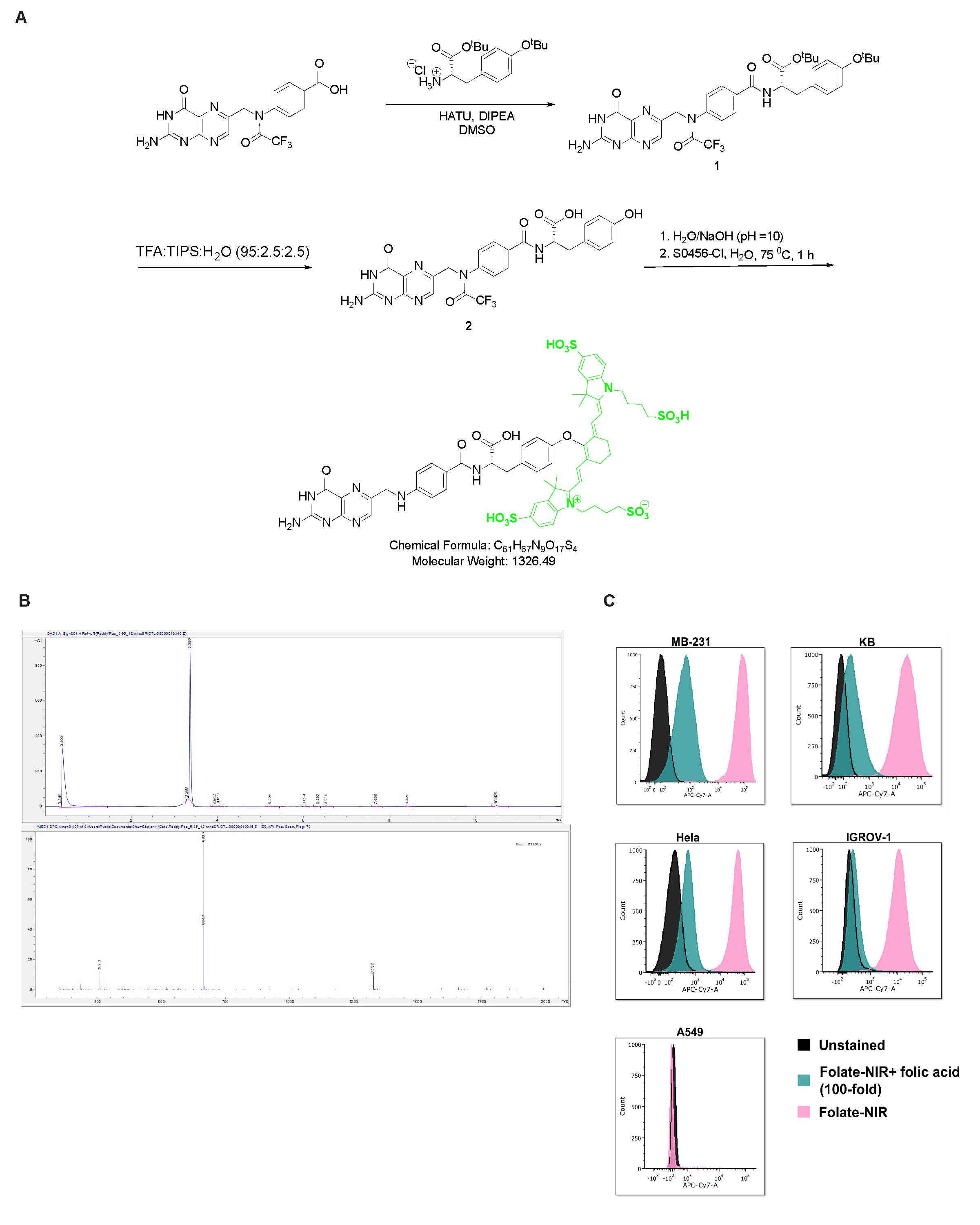


**fig. S6. OTL-38** (**Folate-NIR**) **synthesis, LC-MS spectral analysis and binding to folate receptor (FR) expressing cells. A**, Synthetic scheme for OTL-38. **B**, LC-MS spectrum of OTL-38. **C**, Assessment of folate-NIR binding to FR expressing cells using flow cytometry (n=3).


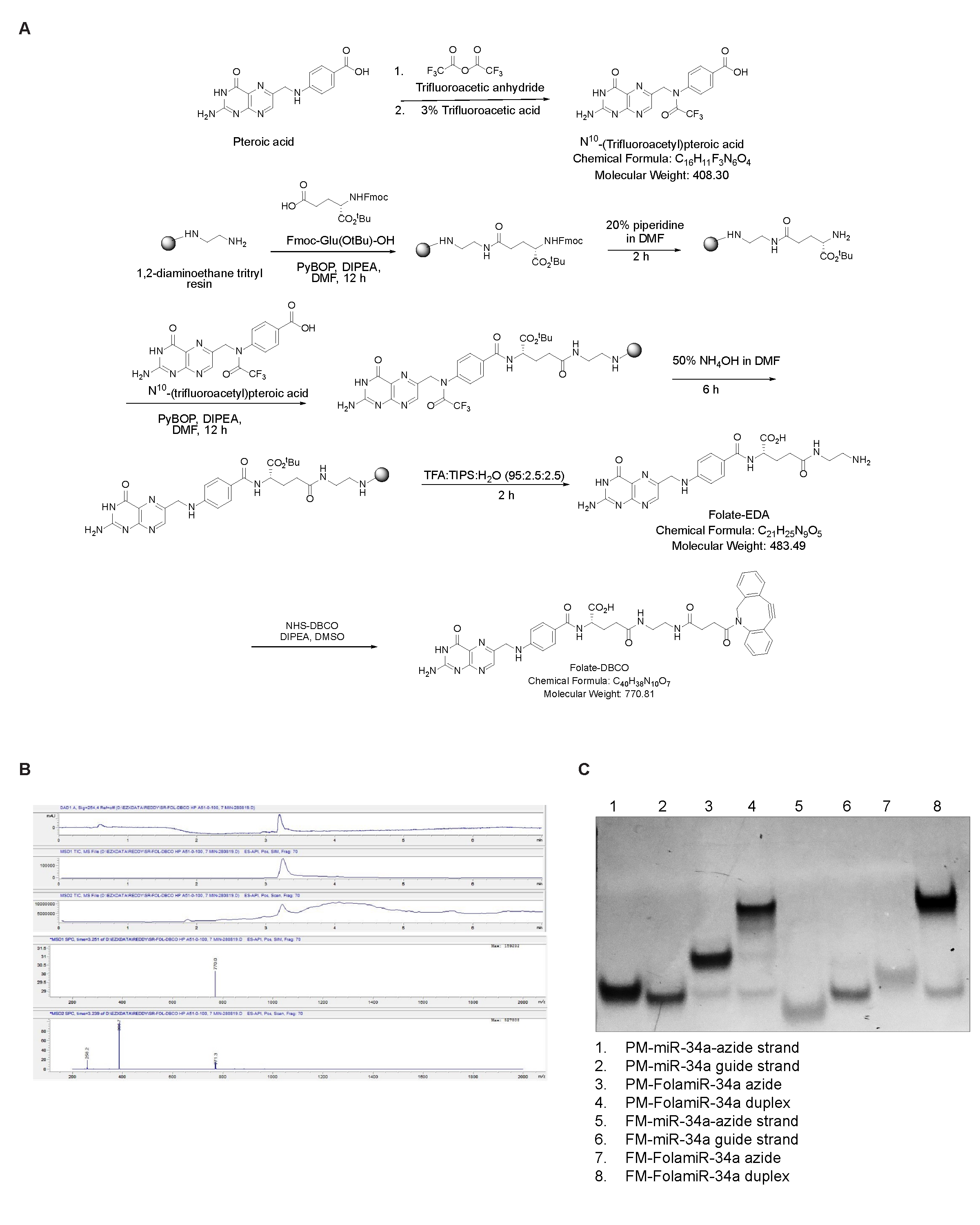


**fig. S7. Folate-DBCO ligand synthesis, LC-MS spectral analysis and validation of PM-FolamiR-34a and FM-FolamiR-34a conjugation. A**, Synthesis of Folate-DBCO by solid phase peptide synthesis method. **B**, LC-MS spectrum of Folate-DBCO conjugate. **C**, Representative gel-Red-stained poly-acrylamide gel of PM and FM-FolamiR-34a shows successful conjugation as indicated by shift in the mobility on the gel.


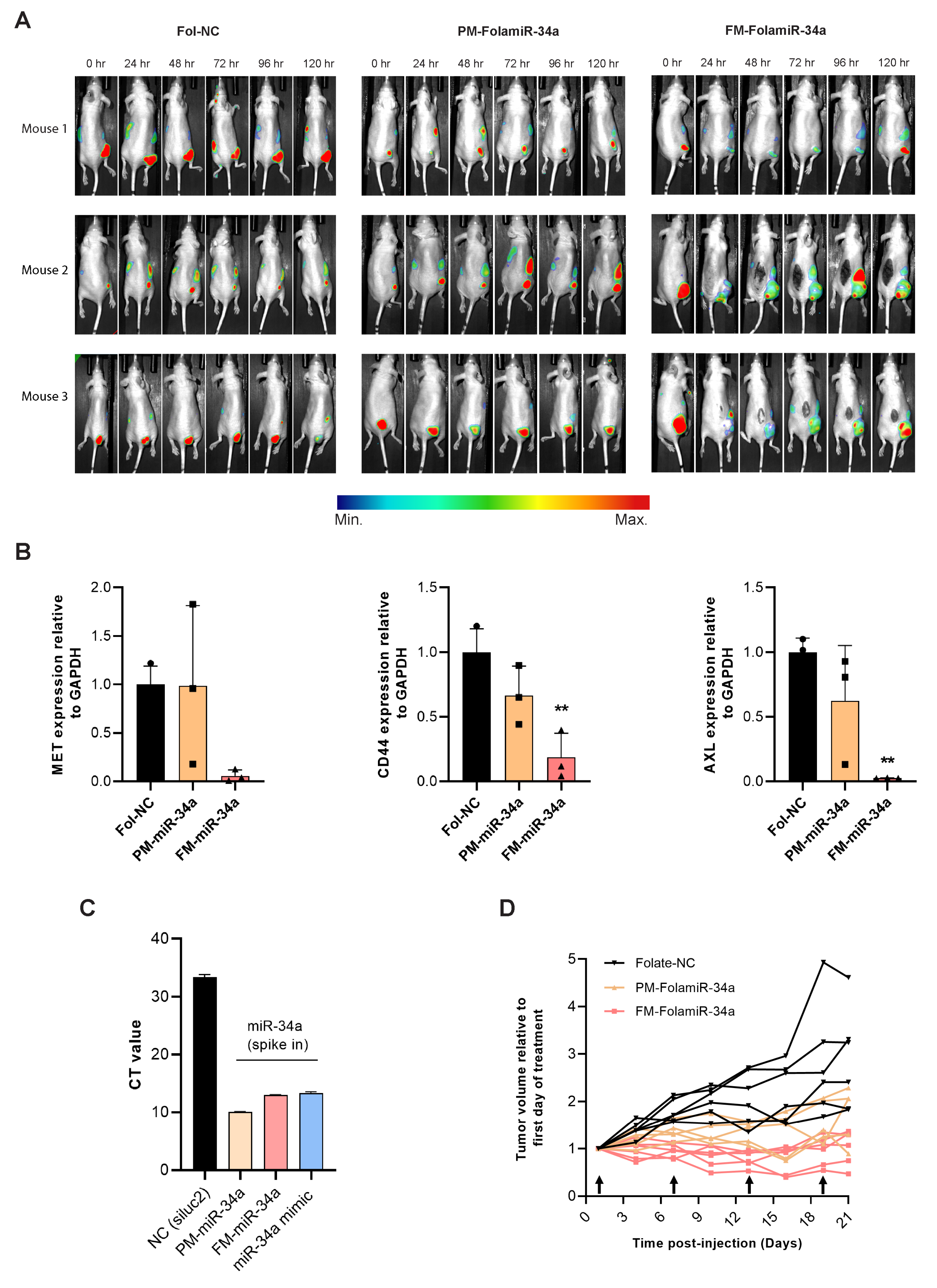
­­

**fig. S8. Effect of FM-FolamiR-34a and PM-FolamiR-34a on MB-231-miR-34a sensor cells and tumor growth *in vivo*. A**, Renilla luciferase imaging of mice bearing MB-231-miR-34a sensor cells after a single intravenous injection of 1.5 nmol of FM-FolamiR-34a, PM-FolamiR-34a, or Fol (folate)-NC. N=3 mice per group. **B**, Quantification of MET, CD44, and AXL expression normalized to GAPDH and relative to Fol-NC (n=3 mice per group; error bars: means ± SD; **p < 0.01, one-way ANOVA with Tukey's multiple comparisons test). Quantificaiton is of western blot depicted in Figure 6. **C**,Confirmation of FM-miR-34a detection using qRT-PCR. An equal amount of FM-miR-34a, PM-miR-34a, or miR-34a mimic was used to make cDNA folllowed by determining CT values using qRT-PCR. Error bars: means ± SD. One experiment is shown of two biological replicates. **D,** Graph of tumor volumes for each mouse treated with the indicated folate-conjugates throughout the study, data was normalized to first day of treatment (folate-NC: n=6 mice , PM-FolamiR-34a: n=5 mice, FM-FolamiR-34a: n=6 mice).

References:

1. Nelson PT, De Planell-Saguer M, Lamprinaki S, Kiriakidou M, Zhang P, O’Doherty U, Mourelatos Z (2007) A novel monoclonal antibody against human Argonaute proteins reveals unexpected characteristics of miRNAs in human blood cells. RNA. https://doi.org/10.1261/rna.646007

2. Orellana EA, Abdelaal AM, Rangasamy L, Tenneti S, Myoung S, Low PS, Kasinski AL (2019) Enhancing MicroRNA Activity through Increased Endosomal Release Mediated by Nigericin. Mol Ther Nucleic Acids 16:505–518
